# Myocarditis and pericarditis associated with SARS-CoV-2 vaccines: A population-based descriptive cohort and a nested self-controlled risk interval study using electronic health care data from four European countries

**DOI:** 10.3389/fphar.2022.1038043

**Published:** 2022-11-24

**Authors:** Sophie H. Bots, Judit Riera-Arnau, Svetlana V. Belitser, Davide Messina, Maria Aragón, Ema Alsina, Ian J. Douglas, Carlos E. Durán, Patricia García-Poza, Rosa Gini, Ron M. C. Herings, Consuelo Huerta, Malede Mequanent Sisay, Mar Martín-Pérez, Ivonne Martin, Jetty A. Overbeek, Olga Paoletti, Meritxell Pallejà-Millán, Anna Schultze, Patrick Souverein, Karin M. A. Swart, Felipe Villalobos, Olaf H. Klungel, Miriam C. J. M. Sturkenboom

**Affiliations:** ^1^ Division of Pharmacoepidemiology and Clinical Pharmacology, Utrecht Institute for Pharmaceutical Sciences, Utrecht University, Utrecht, Netherlands; ^2^ Department of Datascience and Biostatistics, Julius Center for Health Sciences and Primary Health, University Medical Center Utrecht, Utrecht, Netherlands; ^3^ Clinical Pharmacology Service, Vall d’Hebron Hospital Universitari, Vall d’Hebron Barcelona Hospital Campus, Universitat Autònoma de Barcelona, Barcelona, Spain; ^4^ Agenzia Regionale di Sanitá, Florence, Toscana, Italy; ^5^ Fundació Institut Universitari per a la recerca a l’Atenció Primària de Salut Jordi Gol i Gurina (IDIAPJGol), Barcelona, Spain; ^6^ Faculty of Epidemiology and Population Health, London School of Hygiene & Tropical Medicine, London, United Kingdom; ^7^ Spanish Agency for Medicines and Medical Devices (AEMPS), Madrid, Spain; ^8^ PHARMO Institute for Drug Outcomes Research, Utrecht, Netherlands; ^9^ Unitat de Suport a la Recerca Tarragona-Reus, Fundació Institut Universitari per a la recerca a l’Atenció Primària de Salut Jordi Gol i Gurina (IDIAPJGol), Barcelona, Spain

**Keywords:** myocarditis, pericarditis, COVID-19 vaccine, adverse drug reaction, pharmacovigilance

## Abstract

**Background:** Estimates of the association between COVID-19 vaccines and myo-/pericarditis risk vary widely across studies due to scarcity of events, especially in age- and sex-stratified analyses.

**Methods:** Population-based cohort study with nested self-controlled risk interval (SCRI) using healthcare data from five European databases. Individuals were followed from 01/01/2020 until end of data availability (31/12/2021 latest). Outcome was first myo-/pericarditis diagnosis. Exposures were first and second dose of Pfizer, AstraZeneca, Moderna, and Janssen COVID-19 vaccines. Baseline incidence rates (IRs), and vaccine- and dose-specific IRs and rate differences were calculated from the cohort The SCRI calculated calendar time-adjusted IR ratios (IRR), using a 60-day pre-vaccination control period and dose-specific 28-day risk windows. IRRs were pooled using random effects meta-analysis.

**Findings:** Over 35 million individuals (49·2% women, median age 39–49 years) were included, of which 57·4% received at least one COVID-19 vaccine dose. Baseline incidence of myocarditis was low. Myocarditis IRRs were elevated after vaccination in those aged < 30 years, after both Pfizer vaccine doses (IRR = 3·3, 95%CI 1·2-9.4; 7·8, 95%CI 2·6-23·5, respectively) and Moderna vaccine dose 2 (IRR = 6·1, 95%CI 1·1-33·5). An effect of AstraZeneca vaccine dose 2 could not be excluded (IRR = 2·42, 95%CI 0·96-6·07). Pericarditis was not associated with vaccination.

**Interpretation:** mRNA-based COVID-19 vaccines and potentially AstraZeneca are associated with increased myocarditis risk in younger individuals, although absolute incidence remains low. More data on children (≤ 11 years) are needed.

## Introduction

In July 2021, the European Medicines Agency’s (EMA) Pharmacovigilance Risk Assessment Committee (PRAC) announced that mRNA-based COVID-19 vaccines may elevate the risk of myo- and pericarditis (EMA press office, 2021) and this was added to the vaccine’s Summary of Product Characteristics (European Medicines Agency, 2021a; European Medicines Agency, 2021b). By August 2022, approximately 1,2 billion COVID-19 vaccine doses had been distributed within the European population, of which 76% were mRNA-platform based (European Centre for Disease Prevention and Control, 2022). Current evidence of the association between COVID-19 vaccines and myocarditis comprised many case reports and case series (Lee et al., 2022) and six observational studies (Barda et al., 2021; Husby et al., 2021; Le Vu et al., 2021; Patone et al., 2021; Witberg et al., 2021; Karlstad et al., 2022). These suggest an elevated risk of myocarditis and potentially pericarditis in the first 7–14 days after vaccination, especially in younger men after the second dose. Most of the cases resolved without problems (EMA press office, 2021; Klamer et al., 2022).

Uncertainty remains around dose, age, sex, and platform effects as two observational studies do not provide data by vaccine dose (Barda et al., 2021; Husby et al., 2021) and three focussed on mRNA-based COVID-19 vaccines only (Barda et al., 2021; Husby et al., 2021; Le Vu et al., 2021). The largest study up to date was limited in their ability to assess effects of the adenovirus-based COVID-19 vaccines because they used data from Nordic countries where vaccine strategies relied almost exclusively on mRNA-based vaccines (Karlstad et al., 2022). Moreover, none of the studies were able to evaluate the effect of the Janssen vaccine.

This study reports myocarditis and pericarditis incidence rates (IRs) before and after vaccination with four EMA-approved COVID-19 vaccines and assesses the association between each vaccine dose and myo- and pericarditis risk using electronic health record data from four European countries.

## Methods

### Databases

We used data from five European data sources: the Dutch PHARMO Database Network (NL-PHARMO) (Kuiper et al., 2020), the Spanish *Base de Datos para la Investigación Farmacoepidemiológica en Atención Primaria* (ES-BIFAP) database (Maciá-Martínez et al., 2020), the Spanish *Sistema d’Informació per el Desenvolupament de la Investigació en Atenció Primària* (ES-SIDIAP), the Italian *Agenzia Regionale Di Sanità della Toscana* (IT-ARS) database, and the British Clinical Practice Research Datalink (UK-CPRD) Aurum (Clinical Practice Research Datalink, 2022). For more details see Table 1.

**TABLE 1:** Characteristics of the participating data sources.

	IT-ARS	ES-BIFAP	ES-BIFAP-HOSP	ES-SIDIAP	NL-PHARMO	UK-CPRD
Geographical location	Italy, Tuscany region	Spain, multiple regions	Spain, multiple regions	Spain, Catalonia region	Netherlands	United Kingdom
National Population coverage	100%	9%	9%	80%	45%	20%
End of data availability	August 2021	October 2021	October 2021	June 2021	December 2021	December 2021
Myo- and pericarditis diagnoses	Discharge diagnoses & emergency visits	General practice & specialist information reported back	General practice & discharge diagnoses	General practice & discharge diagnoses	General practice & specialist information reported back	General practice & specialist information reported back
Diagnosis coding system	ICD9CM	SNOMED, ICD9CM	ICD10CM	IC10CM	ICPC*	SNOMED, RCCD2
COVID-19 vaccination	Immunisation register	COVID-19 vaccination register	COVID-19 vaccination register	Clinical records	GP records	GP records
COVID-19 diagnoses	COVID-19 PCR-RT register	PCR-RT tests	PCR-RT tests	PCR-RT tests	Diagnoses & PCR-RT tests	GP records
Relevant linked information sources	In- and outpatient pharmacy dispensing, diagnostic tests and procedures, mortality register, mental health service register	Pharmacy dispensing	Pharmacy dispensing	Community pharmacy invoices, specialist referrals		

*This coding system does not differentiate between myocarditis and pericarditis, GP: general practitioner.

### Study populations and design

This study combines a retrospective cohort and a nested self-controlled risk interval (SCRI) study. For the cohort, all registered individuals who had at least 365 days of data availability prior to 1 January 2020 were eligible for inclusion. Follow-up ended at the first myo-/pericarditis diagnosis, death, or end of data availability, whichever came first. Person-time was categorised into non-vaccinated and vaccinated post dose 1 or dose 2. Dose-specific follow-up was 28 days but was censored if administration of the next vaccine dose happened within this interval.

The SCRI design is a modification of a self-controlled case series (SCCS), which uses a fixed control window set before the first vaccine dose (Baker et al., 2015). For the SCRI, all individuals enrolled in the study cohort with at least one COVID-19 vaccine dose and a myo-/pericarditis diagnosis during follow-up were eligible for inclusion. Patients with a heterologous second dose (0.81%) or unknown brand were excluded since the group was too small to look at separately and to avoid misclassification of brands. Data from ES-BIFAP-HOSP were excluded to avoid duplicating cases from ES-BIFAP. Per design, only cases occurring between the start of the control window and the end of the study period were included. The 60-day control window started 90 days before the first vaccine dose, and 28-day risk windows were defined after each vaccine dose. If the second dose was administered within the first dose risk window, the second dose took precedence. To account for potential event-dependency of the exposure, a pre-exposure buffer window starting 30 days prior to the first vaccine dose was included (Supplementary Figure S1).

### Exposure

The exposures of interest were the first and second dose the Pfizer/BioNtech (Comirnaty), Moderna (Spikevax), AstraZeneca (Vaxzevria), and Janssen (COVID-19 Vaccine Janssen) vaccines.

### Outcomes

The main outcomes were a recorded diagnosis of myocarditis or pericarditis, defined by using data source-specific code lists (Supplementary Table S2). Individuals experiencing both events contributed to both the myocarditis and pericarditis analysis.

### Covariates

We extracted information on age, sex, and COVID-19 disease (diagnosis or PCR-RT test). We also extracted information on diagnoses of cardiovascular disease, cancer, chronic lung disease, HIV, chronic kidney disease, diabetes (type 1 and 2), severe obesity, sickle cell disease, and the use of immunosuppressants prior to cohort entry. Each condition was defined based on a combination of diagnosis codes and proxy medications (Willame et al., 2021). The cohort used age groups 5-11, 12-17, 18-29, and ≥ 30 years. The SCRI used < 30 and ≥30 years due to the scarcity of events in younger age groups.

### Data management and quality control

All five data sources converted their local data to the Conception common data model (Thurin et al., 2022) resulting in structurally harmonised local datasets that were quality checked (Hoxhaj, 2022a; van den Bor, 2022; Hoxhaj, 2022b). Analyses were performed locally, and the aggregated output was uploaded to a Digital Research Environment for standardisation and meta-analysis.

### Statistical analyses

Descriptive characteristics are given as mean (standard deviation), median [interquartile range], or number (percentage). Incidence rates (IR) were calculated with the number of cases as numerator and person-years as denominator. Confidence intervals (CI) were calculated using exact methods (Ulm, 1990). For persons aged ≥ 30 years, age-standardised rate differences were calculated by subtracting the age-standardised background myo-pericarditis incidence from the age-standardised incidence after each vaccination instance using the *dsr* package with the Eurostat population as reference. Due to heterogeneity in vaccine programmes targeted at children, incidence rate differences were calculated using the *fmsb* package (Nakazawa, 2022) and included only persons without COVID-19 diagnosis to avoid confounding.

The SCRI analysis was adjusted for calendar time in 30-day time periods. Analyses are presented for the whole population by outcome type and vaccine brand and dose, as well as stratified by sex and age group. Conditional Poisson regression was implemented using R code based on the *SCCS* package (Dif, 2022) adapted to include multiple doses and vaccine brands. Aggregated data were pooled *via* random effects meta-analysis using the *meta* package (Balduzzi et al., 2019). The assumptions underlying the SCRI design were tested. We performed two sensitivity analyses: 1) excluding those with COVID-19 disease before the control window to check the effect of COVID-19 disease; 2) using weekly risk windows (1–7 days, 8–14 days, 15–21 days, 22–28 days) to check for a potential time-response effect. The 7-day window was chosen based on literature suggesting the majority of myocarditis events occur within a week after vaccination. Both sensitivity analyses were performed in the whole population only. Supplementary material S1 provides the calculation for the number of excess cases.

### Missing data

Individuals with missing data on sex, date of birth, or date of database entry or exit (< 1%) were excluded (Supplementary Table S1). Due to the clinical nature of the data, missing data on exposure or outcome was interpretated as informative missingness and thus coded as absence of exposure or outcome, respectively. Missing data on covariates were not imputed as they were only used for descriptive tables.

### Governance and transparency

The protocol (Sturkenboom, 2021), R scripts for data quality checks (Hoxhaj, 2022a; van den Bor, 2022; Hoxhaj, 2022b) and data transformation are publicly available (Gini, 2022). This study was exempt from ethics review due to its observational nature and anonymized data use. This study was conducted rapidly on request of the European Medicines Agency. There was no direct involvement of patients or members of the public in the design, analysis, or reporting of this study. Main study results were shared and discussed with members of the PRAC and the Medicines and Healthcare Products Regulatory Agency from the United Kingdom.

## Results

### Cohort–baseline incidence and vaccination uptake

The study cohort comprised 35,369,669 persons (49·2% women) with a median age of 39–49 years across the four countries. Approximately 25%–35% of the study population had at least one risk factor for severe COVID-19 disease at the start of the study (Table 2).

**TABLE 2:** Baseline characteristics of the study population for the cohort study at 1 January 2020.

		IT-ARS	ES-BIFAP	ES-BIFAP-HOSP	ES-SIDIAP	NL-PHARMO	UK-CPRD
Study population (*n*, %)		3,490,375 (9.9)	11,996,689 (33.9)	9,211,907*	5,283,944 (14.9)	2,302,018 (6.5)	12,296,643 (34.8)
Time of follow-up (person-years)		5,670,102	19,040,824	9,660,986	7,770,414	3,621,169	21,686,454
Demographic characteristics							
Female person-years (%)		47.9	48.2	47.7	49.3	49.0	50.5
Age in years (median, IQR)		49 [29-66]	44 [26-60]	45 [27-61]	43 [25-59]	44 [23-61]	39 [21-57]
Age categories (%)	0–4	3.3	4.0	3.9	4.2	4.4	5.5
	5–11	6.1	7.1	7.0	7.2	7.2	8.7
	12–17	5.3	6.1	6.0	6.3	6.8	6.9
	18–24	6.1	6.4	6.3	6.8	8.2	8.3
	25–29	4.5	5.0	5.0	5.4	6.0	6.8
	30–39	10.3	13.2	13.2	13.4	12.0	14.5
	40–49	14.9	17.1	17.2	17.4	13.0	13.4
	50–59	16.1	14.8	14.9	14.2	15.2	13.6
	60–69	12.9	11.1	11.3	10.8	13.2	10.0
	70–79	11.5	8.4	8.6	8.3	9.7	7.7
	80+	9.1	6.8	6.7	5.9	4.3	4.6
Risk factors for severe COVID-19 at 1 January 2020 (%)							
Cardiovascular disease		27.8	18.5	18.3	19.1	19.4	17.5
Cancer		2.4	1.6	1.9	2.1	2.2	1.3
Chronic lung disease		5.6	4.9	5.0	6.3	6.2	7.3
HIV		0.3	0.0	0.0	0.0	0.1	0.0
Chronic kidney disease		0.5	0.5	0.5	0.9	0.6	0.2
Diabetes		5.6	5.8	5.6	6.4	4.8	5.0
Severe obesity		0.2	0.9	1.0	2.5	0.2	0.7
Sickle cell disease		0.1	0.1	0.1	0.1	0.0	0.0
Use of immunosuppressants		6.0	1.4	1.4	1.8	2.8	0.4
At least one risk factor		34.4	23.9	23.9	26.2	26.2	24.1

*ES-BIFAP-HOSP, is subpopulation part of ES-BIFAP, therefore the study population percentage has not been calculated. CI: confidence interval, IRR: incidence rate ratio. IRRs, are adjusted for calendar time using 30-day periods, IQR: interquartile range; HIV: human immunodeficiency virus.

**FIGURE 1:**
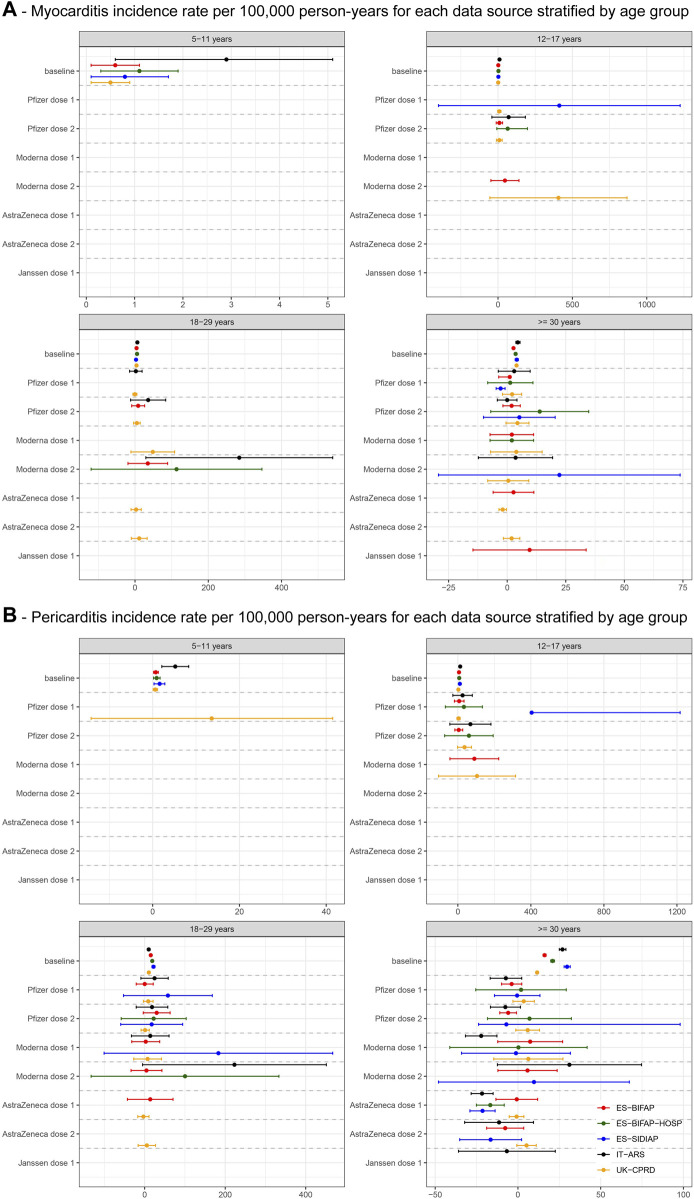
The figure shows crude incidences for the age categories below 30 years and age-standardised rates for the > = 30 years category.

Across data sources, the 2020 background myocarditis incidence in persons without COVID-19 disease ranged from 0·5–2·9/100,000 person-years (PY) in children aged 5–11 years, 1·2-9·9 in persons aged 12–17 years, 2·8–6·4 for persons aged 18–29 years, and 2·7–4·5 for individuals ≥ 30 years (Figure 1A). For pericarditis, these values were 0·6–5·2, 2·8–12·6, 10·0–21·9, and 11·6–29·7, respectively (Figure 1B).

In total, 20,484,323 individuals (57.9%) received at least one COVID-19 vaccine dose, of whom 77·6% received at least two doses (0·81% heterologous) (Table 3). The distribution of vaccine brands varied across countries, with the Pfizer vaccine accounting for two-thirds of first doses in Italy, Spain, and the Netherlands, whereas the AstraZeneca vaccine comprised 50% of first doses in the United Kingdom. Only 7·3% and 2·7% of individuals received the Moderna and Janssen vaccines, respectively. Distance between vaccine doses differed between countries, with median distances being longest in the United Kingdom and shortest in Spain (Table 3). Vaccines brands were channelled to specific population groups based on country, age, and comorbidities (Supplementary Table S3).

**TABLE 3:** Vaccinated persons and distances in days between dose one and for all COVID-19 vaccines.

	Total* *N* = 35,369,669	IT-ARS *N* = 3,490,375	ES-BIFAP *N* = 11,996,689	ES-BIFAP-HOSP *N* = 9,211,907	ES-SIDIAP *N* = 5,283,944	UK-CPRD *N* = 12,296,643	NL-PHARMO *N* = 2,302,018
Total population vaccinated (*n*, %)	20,484,323 (57.9)	2,451,105 (70.2)	6,301,092 (52.5)	574,782 (6.2)^&^	2,793,996 (52.9)	7,466,594 (60.7)	1,471,536 (63.9)
AstraZeneca (Vaxzevria)							
Dose 1 (*n*, %)	5,849,250 (28.6)	333,457 (13.6)	1,025,725 (16.3)	76,841 (13.4)	555,077 (19.9)	3,785,156 (50.7)	149,835 (10.2)
Homologous Dose 2 (%)	82.5	91.7	63.8	96.9	52.4	92.5	48.4
Heterologous Dose 2 (%)	2.5	4.6	1.4	1.1	2.1	2.7	<0.1
Not received Dose 2 (%)	15.0	3.7	34.8	2.0	45.5	4.8	51.2
Time between dose 1 and dose 2 (median, IQR in days)	–	84 [84-84]	79 [74-86]	82 [72-84]	84 [79-92]	77 [69-79]	77 [76-84]
Moderna (Spikevax)							
Dose 1 (*n*, %)	1,488,801 (7.3)	344,899 (14.1)	600,100 (9.5)	76,254 (13.3)	234,851 (8.4)	200,407 (2.7)	108,544 (7.4)
Homologous Dose 2 (%)	77.6	66.1	86.9	91.0	74.0	84.9	57.4
Heterologous Dose 2 (%)	0.2	0.1	0.1	0.1	< 0.1	0.6	<0.1
Not received Dose 2 (%)	22.2	33.8	13.0	9.9	26.0	14.5	42.6
Time between dose 1 and dose 2 (median, IQR in days)	–	42 [28-42]	28 [28-28]	28 [28-28]	28 [28-29]	63 [57-77]	35 [35-35]
Pfizer (Comirnaty)							
Dose 1 (*n*, %)	12,388,919 (60.5)	16,99,855 (69.4)	4,352,441 (69.1)	391,433 (68.1)	1,875,374 (67.1)	34,80,150 (46.6)	981,099 (66.7)
Homologous Dose 2 (%)	78.8	81.3	81.8	93.7	67.4	81.8	71.8
Heterologous Dose 2 (%)	0.1	< 0.1	< 0.1	< 0.1	< 0.1	0.4	< 0.1
Not received Dose 2 (%)	21.2	18.7	18.2	6.3	32.6	17.8	28.2
Time between dose 1 and dose 2 (median, IQR in days)	–	42 [21-42]	21 [21-22]	21 [21-21]	21 [21-22]	74 [59-78]	35 [35-36]
Janssen (Janssen COVID-19 vaccine)							
Dose 1 (n, %)	563,984 (2.7)	72,894 (3.0)	322,588 (5.1)	30,254 (5.3)	128,694 (4.6)	626 (0.0)	39,182 (2.7)
Heterologous Dose 2 (%)	0.4	< 0.1	0.3	0.8	< 0.1	75.2	1.6
Not received Dose 2 (%)	99.6	100	99.7	99.2	100	24.8	98.4
Unknown brand	193,369 (0.9)	–	238 (< 0.1)	–	–	255 (< 0.1)	192,876 (13.1)

*excluding ES-BIFAP-HOSP. ^&^low percentage as most of the regions with hospital data had only data until end of 2020, prior to vaccination. IQR: interquartile range.

### Cohort–incidence rates after COVID-19 vaccination

In children 5–11 years we did not observe any myo- or pericarditis events after COVID-19 vaccination during follow-up (Figure 1; Supplementary Table S4A).

In adolescents aged 12–17 years, the rate difference between post-vaccination and background rates of myocarditis and pericarditis rates was elevated in all data sources after both Pfizer vaccine doses. Non-significant increases in myocarditis rates were seen after Moderna vaccine dose 1 in ES-BIFAP and after dose 2 in UK-CPRD. Follow-up after the AstraZeneca (1,262 PY) and Janssen (62·5 PY) vaccines was limited in this age group and no events were observed after vaccination (Figure 1; Supplementary Table S4A).

In individuals aged 18–29 years, the myocarditis and pericarditis rate differences were elevated after both Pfizer vaccine doses, but not significantly in single data sources. A significant increase in the rate of myocarditis was seen after Moderna vaccine dose 2 in IT-ARS (RD = 284·7, 95%CI 29·6-539·9). Myocarditis IR was also increased after both AstraZeneca vaccine doses in UK-CPRD, but not statistically significant. No events were identified after the Janssen vaccine (763 PY follow-up) (Figure 1; Supplementary Table S4A).

In individuals aged ≥ 30 years, age-standardized pericarditis IRs were significantly decreased after AstraZeneca vaccine dose 1 in IT-ARS (RD = −21·7, −28·3; −15·0), ES-BIFAP-HOSP (RD = −16·7, 95%CI −25·1; −8·3) and ES-SIDIAP (RD = −2·8, 95%CI −4·7; −0·9). We also observed lower pericarditis rates after Pfizer vaccine dose 2 in ES-BIFAP (RD = −5·9, 95%CI −11·0; −0·9) and Moderna vaccine dose 1 in IT-ARS (RD = −22·2, 95%CI −31·7; −12·7). Myocarditis rates were only slightly elevated, and with heterogeneity across data sources (Figure 1; Supplementary Table S4B). In PHARMO standardised myopericarditis IRs were significantly decreased after Moderna vaccine dose 1 (RD = −21·2, 95%CI −23·5; −18·9) and AstraZeneca vaccine dose 2 (RD = −21·2, 95%CI −23·5; −18·9) (Supplementary Table S4C).

### Self-controlled risk interval analyses

In total, 2,703 myo-/pericarditis cases were eligible for inclusion in the SCRI. UK-CPRD contributed most cases (47%), and the majority of cases received the Pfizer vaccine (57%). Twenty-five cases from NL-PHARMO were excluded because vaccine brand was unknown and three cases were censored between the end of the pre-exposure window and the start of the first risk window, resulting in a total of 2,675 myo- or pericarditis cases included in the analyses. As the records from NL-PHARMO (*n* = 115) could not be separated into myo- and pericarditis, the outcome-specific analyses included 2,560 cases (492 myocarditis, 2,124 pericarditis, 56 both).

Pooled myocarditis incidence rate ratios (IRRs) were significantly increased after the 2^nd^ dose of the Pfizer (IRR = 3·18, 95%CI 1·65-6·12) and Moderna (IRR = 5·28, 95%CI 1·68-16·6) vaccines in the whole population (Table 4). The same trend was observed for AstraZeneca vaccine dose 2, albeit not statistically significant (IRR = 2·42, 95%CI 0·96-6·07). Stratification by age showed that the Pfizer and Moderna vaccine effects were driven by individuals aged < 30 years (Table 4). In this age group, myocarditis risk was increased for both doses of the Pfizer vaccine (IRR = 3·30, 95%CI 1·16-9·37; IRR = 7·78, 2·58-23·5, respectively) and Moderna vaccine dose 2 (IRR = 6·05, 95%CI 1·09-33·5). The effect of the Pfizer vaccine was seen in both sexes although the number of cases in women was very low. For the Moderna vaccine, the effect could not be estimated separately in women due to lack of events (Supplementary Table S6). The excess number of cases per 1 million vaccinated individuals was 32·3 (95%CI 10·6-87·0) for the Pfizer vaccine and 33·7 (95%CI 3·8-153·0) for the Moderna vaccine.

**TABLE 4:** Pooled calendar-time adjusted incidence rate ratios for myocarditis and pericarditis from the self-controlled risk interval analysis, stratified by vaccine brand, dose, age, and sex.

	Myocarditis				Pericarditis			
COVID-19 Vaccinations	Unexposed/exposed cases	First dose IRR (95% CI)	Unexposed/exposed cases	Second dose IRR (95% CI)	Unexposed/exposed cases	First dose IRR (95% CI)	Unexposed/exposed cases	Second dose IRR (95% CI)
Whole population								
Control		reference		reference		reference		reference
Pfizer	51/28	1.72 (0.96–3.09)	46/44	3.18 (1.65–6.12)	391/126	0.95 (0.74–1.22)	323/118	0.90 (0.67–1.21)
AstraZeneca	37/11	0.96 (0.42–2.17)	29/17	2.42 (0.96–6.07)	143/58	0.93 (0.50–1.73)	119/55	1.22 (0.57–2.61)
Moderna	14/7	4.00 (0.54–29.5)	11/13	5.28 (1.68–16.6)	68/25	0.99 (0.48–2.04)	57/21	1.11 (0.32–3.83)
Janssen	< 5/< 5	1.59 (0.12–21.6)			20/< 5	0.74 (0.16–3.42)		
12–29 years								
Control		reference		reference		reference		reference
Pfizer	9/10	3.30 (1.16–9.37)	5/14	7.78 (2.58–23.5)	41/26	1.73 (0.98–3.05)	30/22	1.74 (0.98–3.08)
AstraZeneca	7/<5	0.32 (0.04–2.63)	6/<5	0.72 (0.14–3.69)	9/<5	1.14 (0.27–4.81)	8/<5	1.55 (0.41–5.87)
Moderna	<5/<5	3.49 (0.59–20.5)	<5/10	6.05 (1.09–33.5)	11/9	1.84 (0.74–4.58)	8/5	0.48 (0.05–4.18)
Janssen					<5/<5			
≥ 30 years								
Control		reference		reference		reference		reference
Pfizer	42/18	0.90 (0.43–1.89)	41/30	1.34 (0.70–2.59)	350/100	0.75 (0.57–0.98)	293/96	0.67 (0.49–0.91)
AstraZeneca	30/10	0.82 (0.35–1.91)	23/15	1.92 (0.79–4.68)	134/55	0.83 (0.46–1.50)	111/51	0.88 (0.51–1.52)
Moderna	11/<5	0.68 (0.05–9.01)	9/< 5	0.26 (0.04–1.76)	57/16	0.72 (0.22–2.38)	49/26	0.81 (0.42–1.54)
Janssen	< 5/< 5	0.88 (0.07–11.7)			20/<5	0.63 (0.16–2.53)		
Women								
Control		reference		reference		reference		reference
Pfizer	19/10	1.03 (0.43–2.48)	19/13	1.54 (0.67–3.58)	162/49	082 (0.59–1.15)	139/43	0.69 (0.47–1.00)
AstraZeneca	15/<5	0.17 (0.02–1.34)	12/6	1.29 (0.45–3.68)	60/25	0.98 (0.44–2.19)	45/21	1.19 (0.51–2.77)
Moderna	<5/<5		<5/<5		30/6	0.33 (0.11–0.96)	28/8	0.83 (0.36–1.94)
Janssen	<5/<5				11/<5			
Men								
Control		reference		reference		reference		reference
Pfizer	32/18	1.90 (0.98–3.67)	27/31	2.78 (1.53–5.07)	229/77	1.02 (0.70–1.50)	184/75	1.06 (0.79–1.43)
AstraZeneca	22/10	1.19 (0.52–2.74)	17/11	1.91 (0.81–4.51)	83/33	0.88 (0.58–1.35)	74/34	1.08 (0.69–1.71)
Moderna	10/5	2.86 (0.63–13.1)	7/12	5.80 (1.62–20.7)	38/19	1.75 (0.96–3.19)	29/13	1.51 (0.48–4.75)
Janssen	<5/<5	1.24 (0.10–15.7)			9/<5	1.70 (0.33–8.63)		

CI: confidence interval, IRR: incidence rate ratio. IRRs, are adjusted for calendar time using 30-day periods.

There was no significant increase in myocarditis risk in those aged ≥ 30 years (Table 4). Stratification by both age and sex showed that the effect of the AstraZeneca vaccine on myocarditis was strongest in men aged ≥ 30 years (IRR = 8·69, 95%CI 2·54-29·7) (Supplementary Table S6). We did not find evidence for an increased pericarditis risk after vaccination in the whole population (Table 4). Stratification by age and sex showed an isolated finding in men aged < 30 years after Pfizer vaccine dose 2 (Supplementary Table S6). There was no significant association between vaccination and myopericarditis in NL-PHARMO (Supplementary Table S5).

**FIGURE 2:**
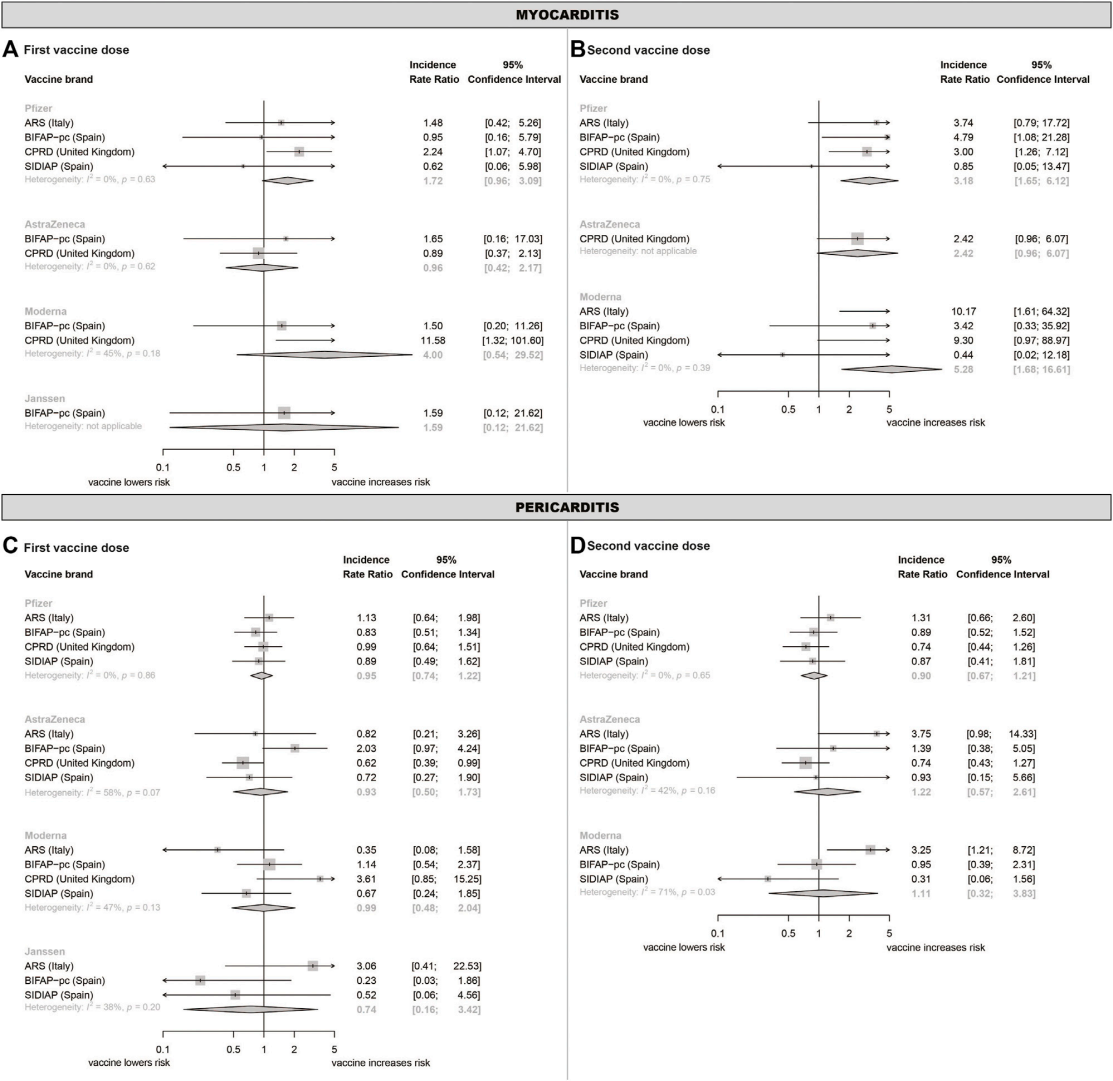
Meta-analysed incidence rate ratios of myo- and pericarditis following COVID-19 vaccination.

Using weekly risk windows suggested myocarditis risk was highest 1–7 days after vaccination with the Pfizer and Moderna vaccines and 8–14 days after vaccination with the AstraZeneca vaccine (Supplementary Table S7). Excluding individuals with prior COVID-19 did not markedly change our conclusions (Supplementary Figure S2). There was some heterogeneity across data sources (Figure 2).

## Discussion

This study, including over 35 million individuals from four European countries, shows that myocarditis rates are elevated in individuals aged < 30 years after both doses of the Pfizer vaccine and the second dose of the Moderna vaccine. We could not exclude an effect of AstraZeneca vaccine dose 2 on myocarditis, but this was mostly based on United Kingdom data where AstraZeneca vaccine was used in younger persons. The Janssen vaccine was not associated with myocarditis incidence, but we had insufficient power due to limited distribution of this vaccine. Importantly, the absolute incidence of myocarditis was low resulting in a low number of excess myocarditis cases per million vaccinated. Pericarditis risk was not significantly elevated after any of the COVID-19 vaccines.

Our results are in line with previous work, although estimates vary strongly between studies. An Israeli target trial using data from a payer-provider healthcare organisation found an increase myocarditis risk within 42 days after the Pfizer vaccine (RR 3·24, 95%CI 1·55–12·44) and no effect on pericarditis (RR 1·27, 95% 0·68–2·31) (Barda et al., 2021). A Danish nationwide cohort study found no statistically significant elevation of myocarditis risk within 27 days after the Pfizer vaccine (HR 1·34, 95%CI 0·90–2·00) (Husby et al., 2021), but did not distinguish by dose. A cohort from four Nordic countries (including Denmark) did find an elevated (but smaller) risk within 28 days after the first (IRR = 1·38, 95%CI 1·12–169) and second (IRR = 1·75, 95%CI 1·43–2·14) dose of the Pfizer vaccine (Karlstad et al., 2022). Similarly, a French nested case-control study using nationwide reimbursement data found elevated myocarditis risk in the 7 days after both the first (OR 1·66, 95%CI 1·16–2·37) and second (OR 6·88, 95%CI 5·28–8·96) Pfizer vaccine dose (Le Vu et al., 2021). A self-controlled case series using the English national immunisation database also showed elevated myocarditis risk after both Pfizer vaccine doses after 1–7 and 1–28-day risk windows, although the effect sizes were closer to the Danish than the French study (Patone et al., 2021). All five studies situated in Europe found increased myocarditis risk after the Moderna vaccine with estimates ranging from approximately 4-fold in the Danish study to almost 26-fold in the French (Barda et al., 2021; Husby et al., 2021; Le Vu et al., 2021; Patone et al., 2021; Karlstad et al., 2022). The literature also supports our observation that myocarditis risk was most strongly increased in those aged 12–29 years, although estimates varied strongly here as well. The length of the risk window may play a role, as our estimates for the Moderna vaccine were much higher when we looked only at the first 7 days after vaccination compared to the 28-day risk interval. For the age-specific analyses, different definitions of the age groups may also explain some of the variation in the estimates.

We could not exclude an increase in myocarditis IR after AstraZeneca vaccine dose 2, although it is difficult to tease out age- and sex-specific effects because this vaccine was predominantly administered to older individuals except in the United Kingdom. The association between the AstraZeneca vaccine and myocarditis risk has only been observed in the English study (Patone et al., 2021), which reported an increase in risk only after the first dose for both a 7-day (IRR = 1·76, 95%CI 1·29–2·42) and a 28-day (IRR = 1·29, 95%CI 1·05–1·58) risk window. We are the first to study the association between the Janssen vaccine and myocarditis, but we had limited power to detect an effect due to low uptake of this vaccine.

COVID-19 vaccines are highly effective in preventing severe COVID-19 disease and are therefore a pivotal means for preventing mortality and morbidity in this pandemic. However, accurate quantification of the risk of rare side effects is crucial to be able to accurately establish benefit-risk profiles of each of the COVID-19 vaccines in specific age groups. Younger people are generally at a lower risk of serious consequences of COVID-19, and the benefit risk balance needs to always be weighed carefully (Bilotta et al., 2022). The EMA is responsible for monitoring the benefit risk balance and in their assessment of the mRNA vaccines has stated that the balance remains positive (European Medicines Agency, 2021b).

The main strengths of this study are its size and geographical coverage, including previously unpublished data from four European countries (Italy, Spain, Netherlands, and Denmark). It combines a cohort design to calculate absolute IRs and RDs with a nested SCRI to adjust for unmeasured confounding. The use of the Conception CDM and pipeline enables streamlined data quality checks and local individual-level analysis. Our results allow for direct comparison between brand within one country and across countries within one brand, showing consistent findings across data sources. Lastly, the validity of the SCRI relies on three important assumptions: 1) event-independent exposure; 2) no event-dependent censoring of observation time; and 3) events are recurrent and independent. We showed that the risk of myocarditis was similar in the control window and the 30-day pre-exposure window, suggesting assumption (1) holds true. The other two assumptions should also hold true because mortality following myo-/pericarditis is low and both events are rare, evidenced by the low absolute incidence rates. There are some limitations to address as well. First, there was a lag in data availability because data available for researchers was updated only periodically. To ensure that all data sources were able to run required quality checks, we truncated follow-up at December 2021. However, some data providers had limited resources to update their data instances. For example, the data instance for ES-SIDIAP was locked in June 2021. Whenever new data become available, we may update our analyses and include additional data sources. Moreover, vaccine exposure was limited in children aged 5–11 years so we could not provide an estimate for this population with the current data instances. Future updates of data may give more insight. Second, we may have underestimated the background rates of myo-/pericarditis due to reduced access to care during lockdown periods, although previous work suggests this effect was minimal (Willame et al., 2021). This does not impact the SCRI as much since the control window was close to the risk windows, minimising differences in baseline rate due to changes in national COVID-19 policy. Third, data sources relying on GP data will record information from specialist and hospitals with some delay. We censored these data sources at December 2021 to minimise this, however we may still have missed some events and the cohort study may therefore have underestimated incidence rates. Fourth, we had limited power to estimate the effects of the AstraZeneca vaccines in persons aged below 30 and the Janssen vaccine in general due to very low use of those vaccines. Future data instances may solve the former, but the latter is difficult to remedy because vaccine programmes simply did not distribute high quantities of Janssen in the countries included in our study.

Myocarditis incidence is elevated in individuals aged < 30 years after both doses of the Pfizer vaccine and the second dose of the Moderna vaccine. AstraZeneca vaccine dose 2 may also have an effect. Additional data on young children (5–11 years) and adenovirus platform vaccines in younger individuals (< 30 years) are needed. Comparison between published studies was complicated by large variation in the definition of risk window length and age groups. Given that COVID-19 disease also poses a risk of life-threatening complications in children, it is paramount to endorse vaccine benefit-risk assessment studies in this population. Future studies focusing on younger age groups should consider harmonising these definitions to facilitate meta-analysis efforts. The absolute incidence of myocarditis remains low and the number of excess cases per million vaccinated are around 30–35 per million vaccinated, supporting the EMA decision that the benefit of COVID-19 vaccination outweighs the risk of myocarditis in younger persons (EMA press office, 2021).

## Data Availability

The data analyzed in this study is subject to the following licenses/restrictions: The local data used for this study are not publicly available. The protocol and scripts for data quality checks, data transformation, and analysis are publicly available on GitHub: https://github.com/VAC4EU/CVM/tree/Release_myocarditis_manuscript.
