# Supplementary material for: Myocarditis and pericarditis associated with SARS-CoV-2 vaccines: A population-based descriptive cohort and a nested self-controlled risk interval study using electronic health care data from four European countries
Source: Front Pharmacol. 2022 Nov 24;13:1038043. doi: 10.3389/fphar.2022.1038043 (PMC9730238; doi:10.3389/fphar.2022.1038043)
Supplement: Supplementary file 1 [file Table8.DOCX]

**Supplementary Table 3.** Characteristics of the study population for the cohort study at first vaccination dose

|  | | IT-ARS | | | | ES-BIFAP | | | | | ES-SIDIAP | | | | UK-CPRD | | | | | NL-PHARMO | | | | |
| --- | --- | --- | --- | --- | --- | --- | --- | --- | --- | --- | --- | --- | --- | --- | --- | --- | --- | --- | --- | --- | --- | --- | --- | --- |
|  | | **Pfizer** | **Moderna** | **AstraZeneca** | **Janssen** | **Pfizer** | **Moderna** | **AstraZeneca** | **Janssen** | **Unknown** | **Pfizer** | **Moderna** | **AstraZeneca** | **Janssen** | **Pfizer** | **Moderna** | **AstraZeneca** | **Janssen** | **Unknown** | **Pfizer** | **Moderna** | **AstraZeneca** | **Janssen** | **Unknown** |
| Study population (n, %) | | 1699855 (69.4) | 344899 (14.1) | 333457 (13.6) | 72894 (3.0) | 4352441 (69.1) | 600100 (9.5) | 1025725 (16.3) | 322588 (5.1) | 238 (0.0) | 1875374 (67.1) | 234851 8.4) | 555077 (19.9) | 128694 (4.6) | 3480150 (46.6) | 200407 (2.7) | 3785156 (50.7) | 626 (0.0) | 255 (0.0) | 981099 (66.7) | 108544 (7.4) | 149835 (10.2) | 39182 (2.7) | 192876 (13.1) |
| Follow-up between first and second dose (person-years, %) | | 155429 (55) | 31983 (11.3) | 77975 (27.6) | 17239 (6.1) | 279652 (45.1) | 54177 (8.7) | 219841 (35.5) | 66334 (10.7) | 32 (0.0) | 99375 (41) | 17662 (7.3) | 116864 (48.2) | 8358 (3.5) | 719639 (44.3) | 40264 (2.5) | 864878 (53.2) | 190 (0.0) | 121 (0.0) | 118347 (58.5) | 9605 (4.7) | 31572 (15.6) | 2442 (1.2) | 40297 (19.9) |
| *Quarter of first vaccination (n, %)* | | | | | |  |  |  |  |  |  |  |  |  |  |  |  |  |  |  |  |  |  |  |
| 2020 December | | 1584 (0.1) | 0 (0.0) | 0 (0.0) | 0 (0.0) | 16081 (0.4) | 0 (0.0) | 0 (0.0) | 15 (0.0) | 4 (1.7) | 4526 (0.2) | 0 (0.0) | 0 (0.0) | 2 (0.0) | 179117 (5.1) | 1 (0.0) | 184 (0.0) | 147 (23.5) | 120 (47.1) | *NA* | *NA* | *NA* | *NA* | *NA* |
| 2021 January-March | | 248532 (14.6) | 37597 (10.9) | 137408 (41.3) | 1 (0.0) | 819359 (18.8) | 58288 (9.8) | 275530 (26.8) | 726 (0.2) | 103 (43.3) | 385724 (20.6) | 33160 (14.2) | 214080 (38.6) | 242 (0.2) | 1521289 (43.7) | 103 (0.0) | 3154381 (83.3) | 307 (49.0) | 9 (3.5) | 64864 (6.6) | 3191 (2.9) | 33814 (22.6) | 4 (0.0) | 26110 (13.6) |
| 2021 April-June | | 1072114 (63.0) | 146707 (42.5) | 195623 (58.7) | 58593 (80.5) | 2907073 (66.8) | 387377 (64.6) | 744355 (72.6) | 289804 (89.9) | 102 (42.8) | 1485124 (79.2) | 201691 (85.9) | 340997 (61.4) | 128450 (99.8) | 1015819 (29.1) | 144172 (71.9) | 608589 (16.1) | 118 (18.8) | 126 (49.4) | 573324 (58.5) | 52305 (48.2) | 113206 (75.5) | 17264 (44.1) | 111094 (57.6) |
| 2021 July-September | | 377625 (22.2) | 160.595 (46.6) | 426 (0.2) | 14300 (19.7) | 596305 (13.7) | 152934 (25.5) | 5823 (0.6) | 31911 (9.9) | 29 (12.2) | *NA* | *NA* | *NA* | *NA* | 443753 (12.7) | 50383 (25.1) | 19242 (0.5) | 45 (7.2) | 0 (0.0) | 294683 (30.1) | 47319 (43.6) | 2542 (1.7) | 19937 (50.9) | 32777 (16.9) |
| 2021 October-December | | *NA* | *NA* | *NA* | *NA* | 13623 (0.3) | 1501 (0.3) | 17 (0.0) | 132 (0.0) | 0 (0.0) | *NA* | *NA* | *NA* | *NA* | 320172 (9.2) | 5748 (2.8) | 2760 (0.1) | 9 (1.4) | 0 (0.0) | 48228 (4.9) | 5729 (5.4) | 273 (0.2) | 1977 (5.1) | 22895 (11.9) |
| *Demographic characteristics* | | | | | |  |  |  |  |  |  |  |  |  |  |  |  |  |  |  |  |  |  |  |
| Women (person-years, %) | | 81871 (52.7) | 15465 (48.4) | 43460 (55.7) | 8947 (51.9) | 151630 | 29124 | 124739 | 31043 | 20 | 54916 (55.3) | 9326 (52.8) | 64185 (54.9) | 4050 48.5) | 380797 (52.9) | 17021 (42.3) | 444613 (51.4) | 85 (44.7) | 58 (48.3) | 61214 (51.7) | 4612 (48.0) | 16365 (51.8) | 926 (37.9) | 22277 (55.3) |
| Age, years (median, IQR) | | 54 [39-71] | 44 [30-58] | 69 [57-74] | 63 [60-69] | 54 [44-73] | 53 [35-58] | 62 [59-64] | 50 [44-57] | 47 [30-63] | 54 [45-74] | 52 [45-57] | 62 [59-65] | 49 [43-65] | 38 [25-65] | 32 [25-39] | 55 [45-65] | 48 [40-57] | 57 [44-65] | 50 [31-68] | 46 [34-54] | 62 [60-63] | 26 [21-42] | 60 [44-73] |
| Age in categories (n, %) | **< 30 years** | 275064 (16.2) | 85149 (24.6) | 7625 (2.3) | 169 (0.2) | 413690 (9.6) | 110160 (18.4) | 42903 (3.1) | 9253 (2.9) | 57 (23.9) | 46145 (2.5) | 14830 (6.3) | 26229 (4.7) | 4163 (3.2) | 1140410 (32.7) | 79226 (39.5) | 179614 (4.8) | 66 (10.5) | 16 (6.2) | 221728 (22.6) | 20671 (19.1) | 4562 (3.1) | 22917 (58.6) | 24363 (12.6) |
|  | **≥ 30 years** | 1424791 (83.8) | 259750 (75.4) | 325832 (97.7) | 72725 (99.8) | 3938751 (90.4) | 489940 (81.6) | 982822 (96.9) | 313335 (97.1) | 181 (76.1) | 1829229 (97.5) | 220021 (93.7) | 528848 (95.3) | 124531 (96.8) | 2339740 (67.3) | 121181 (60.5) | 3605542 (95.2) | 560 (89.5) | 239 (93.8) | 759371 (77.4) | 87873 (80.9) | 145273 (96.9) | 16265 (41.4) | 168513 (87.4) |
| *Medical risk factors at date of vaccination (n, %)* | | | | | |  |  |  |  |  |  |  |  |  |  |  |  |  |  |  |  |  |  |  |
| Cardiovascular disease | | 665286 (39.1) | 87130 (25.3) | 162289 (48.7) | 35944 (49.3) | 1545555 (35.5) | 149803 (25.0) | 346745 (33.8) | 67055 (20.8) | 63 (26.5) | 672002 (35.8) | 60270 (25.7) | 188993 (10.1) | 33127 (25.7) | 883762 (25.4) | 8361 (4.2) | 1117550 (29.5) | 78 (12.5) | 63 (24.7) | 276762 (28.2) | 18632 (17.2) | 56674 (37.8) | 2022 (5.2) | 69705 (36.1) |
| Cancer | | 61855 (3.6) | 22372 (6.5) | 8868 (2.7) | 1741 (2.4) | 146806 (3.4) | 33068 (5.5) | 30320 (3.0) | 6362 (2.0) | 12 (5.0) | 85629 (4.6) | 32601 (13.9) | 20848 (1.1) | 3336 (2.6) | 94979 (2.7) | 482 (0.2) | 108768 (2.9) | 6 (1.0) | 5 (2.0) | 45653 (4.7) | 3807 (3.5) | 7570 (5.1) | 334 (0.9) | 11841 (6.1) |
| Chronic lung disease | | 143330 (8.4) | 24059 (7.0) | 25865 (7.8) | 5364 (7.4) | 420002 (9.6) | 45935 (7.7) | 88577 (8.6) | 22303 (6.9) | 27 (11.3) | 213875 (11.4) | 22911 (9.8) | 55873 (3.0) | 12322 (9.6) | 369128 (10.6) | 9401 (4.7) | 452217 (11.9) | 40 (6.4) | 23 (9.0) | 94157 (9.6) | 8690 (8.0) | 17948 (12.0) | 1055 (2.7) | 21264(11.0) |
| HIV | | 4935 (0.3) | 2036 (0.6) | 389 (0.1) | 114 (0.2) | 2040 (0.0) | 725 (0.1) | 472 (0.0) | 275 (0.1) | 0 (0.0) | 669 (0.1) | 563 (0.2) | 193 (0.0) | 94 (0.1) | 1738 (0.0) | 28 (0.0) | 2673 (0.1) | 2 (0.3) | 0 (0.0) | 1931 (0.2) | 266 (0.2) | 346 (0.2) | 35 (0.1) | 472 (0.2) |
| Chronic kidney disease | | 15820 (0.9) | 4354 (1.3) | 886 (0.3) | 190 (0.3) | 61089 (1.4) | 6144 (1.0) | 4557 (0.4) | 1249 (0.4) | 0 (0.0) | 57236 (3.1) | 4929 (2.1) | 5314 (0.3) | 1438 (1.1) | 15125 (0.4) | 33 (0.0) | 13658 (0.4) | 0 (0.0) | 1 (0.4) | 14353 (1.5) | 859 (0.8) | 1924 (1.3) | 37 (0.1) | 5423 (2.8) |
| Diabetes | | 141456 (8.3) | 21252 (6.2) | 18735 (5.6) | 4565 (6.3) | 483109 (11.1) | 46689 (7.8) | 114941 (11.2) | 22013 (6.8) | 18 (7.6) | 230700 (12.3) | 21426 (9.1) | 70755 (3.8) | 13786 (10.7) | 260143 (7.5) | 1194 (0.6) | 320801 (8.5) | 13 (2.1) | 16 (6.3) | 71314 (7.3) | 5311 (4.9) | 15194 (10.1) | 456 (1.2) | 17984 (9.3) |
| Severe obesity | | 6159 (0.4) | 1375 (0.4) | 595 (0.2) | 210 (0.3) | 84263 (1.9) | 10216 (1.7) | 19512 (1.9) | 5958 (1.8) | 2 (0.8) | 94877 (5.1) | 12724 (5.4) | 30916 (1.6) | 6908 (5.4) | 43087 (1.2) | 1281 (0.6) | 61294 (1.6) | 5 (0.8) | 6 (2.4) | 5467 (0.6) | 578 (0.5) | 1616 (1.1) | 112 (0.3) | 1155 (0.6) |
| Sickle cell disease | | 2593 (0.2) | 633 (0.2) | 185 (0.1) | 30 (0.0) | 6382 (0.1) | 1277 (0.2) | 1026 (0.1) | 239 (0.1) | 1 (0.4) | 3094 (0.2) | 683 (0.3) | 580 (0.0) | 164 (0.1) | 1050 (0.0) | 3 (0.0) | 1027 (0.0) | 0 (0.0) | 0 (0.0) | 641 (0.1) | 69 (0.1) | 30 (0.0) | 7 (0.0) | 145 (0.1) |
| Use of immunosuppressants | | 225832 (13.3) | 46920 (13.6) | 42688 (12.8) | 10328 (14.2) | 146524 (3.4) | 24196 (4.0) | 29817 (2.9) | 7527 (2.3) | 12 (5.0) | 70376 (3.8) | 21238 (9.0) | 16377 (0.9) | 3322 (2.6) | 27373 (0.8) | 218 (0.1) | 36959 (1.0) | 1 (0.2) | 0 (0.0) | 57496 (5.9) | 4985 (4.6) | 11147 (7.4) | 471(1.2) | 14603 (7.6) |
| At least one risk factor | | 823701 (48.5) | 126942 (36.8) | 189089 (56.7) | 42305 (58.0) | 1902334 (43.7) | 207732 (34.6) | 447853 (43.7) | 96086 (29.8) | 90 (37.8) | 855349 (45.6) | 97435 (41.5) | 253684 (13.5) | 48173 (37.4) | 1204405 (34.6) | 19304 (9.6) | 1532361 (40.5) | 123 (19.6) | 87 (34.1) | 375928 (38.3) | 30301 (27.9) | 74821 (49.9) | 3767 (9.6) | 92388 (47.9) |
