# Supplementary material for: Myocarditis and pericarditis associated with SARS-CoV-2 vaccines: A population-based descriptive cohort and a nested self-controlled risk interval study using electronic health care data from four European countries
Source: Front Pharmacol. 2022 Nov 24;13:1038043. doi: 10.3389/fphar.2022.1038043 (PMC9730238; doi:10.3389/fphar.2022.1038043)
Supplement: Supplementary file 2 [file Table12.DOCX]

Supplementary table 5: Pooled incidence rate ratios of myopericarditis in the whole population of PHARMO for the first and second vaccine dose per vaccine brand

|  | **Myopericarditis** | | | |
| --- | --- | --- | --- | --- |
|  | **Individuals**  **(exposed cases)** | **First dose**  **IRR (95% CI)** | **Individuals**  **(exposed cases)** | **Second dose**  **IRR (95% CI)** |
| Whole population  Control  Pfizer  AstraZeneca  Moderna  Janssen | 79 (10)  22 (<5)  10 (<5)  < 5 (<5) | *reference*  1.29 (0.53-3.67)  1.02 (0.23-4.49)  0.62 (0.06-6.04) | 43 (6)  9 (<5)  7 (<5) | *reference*  2.04 (0.54-7.65)  3.5 (0.44-27.6) |
