# Supplementary figures and images for: Myocarditis and pericarditis associated with SARS-CoV-2 vaccines: A population-based descriptive cohort and a nested self-controlled risk interval study using electronic health care data from four European countries
Source: Front Pharmacol. 2022 Nov 24;13:1038043. doi: 10.3389/fphar.2022.1038043 (PMC9730238; doi:10.3389/fphar.2022.1038043)

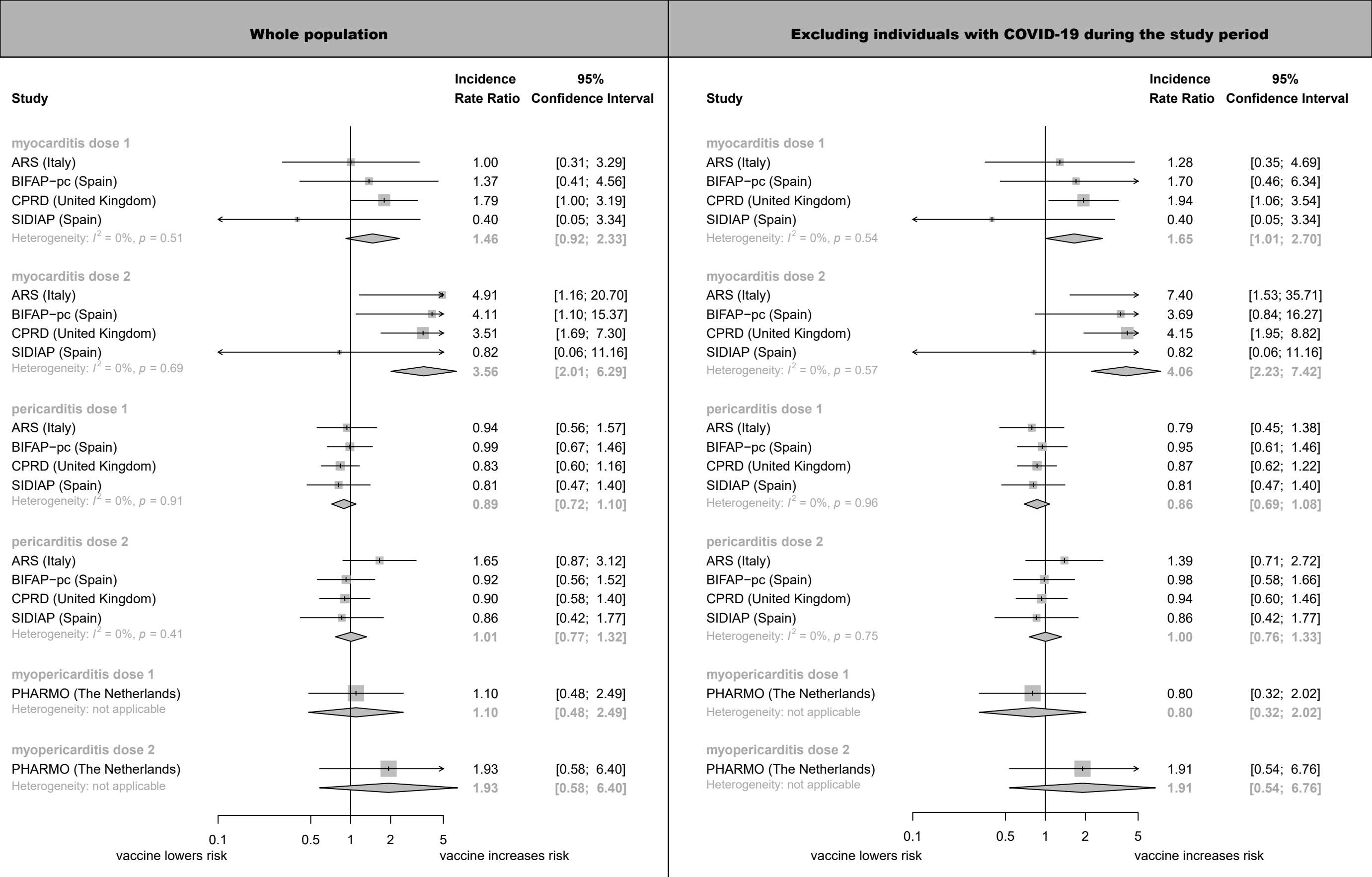

Supplement: Supplementary file 3 [file Image2.pdf]

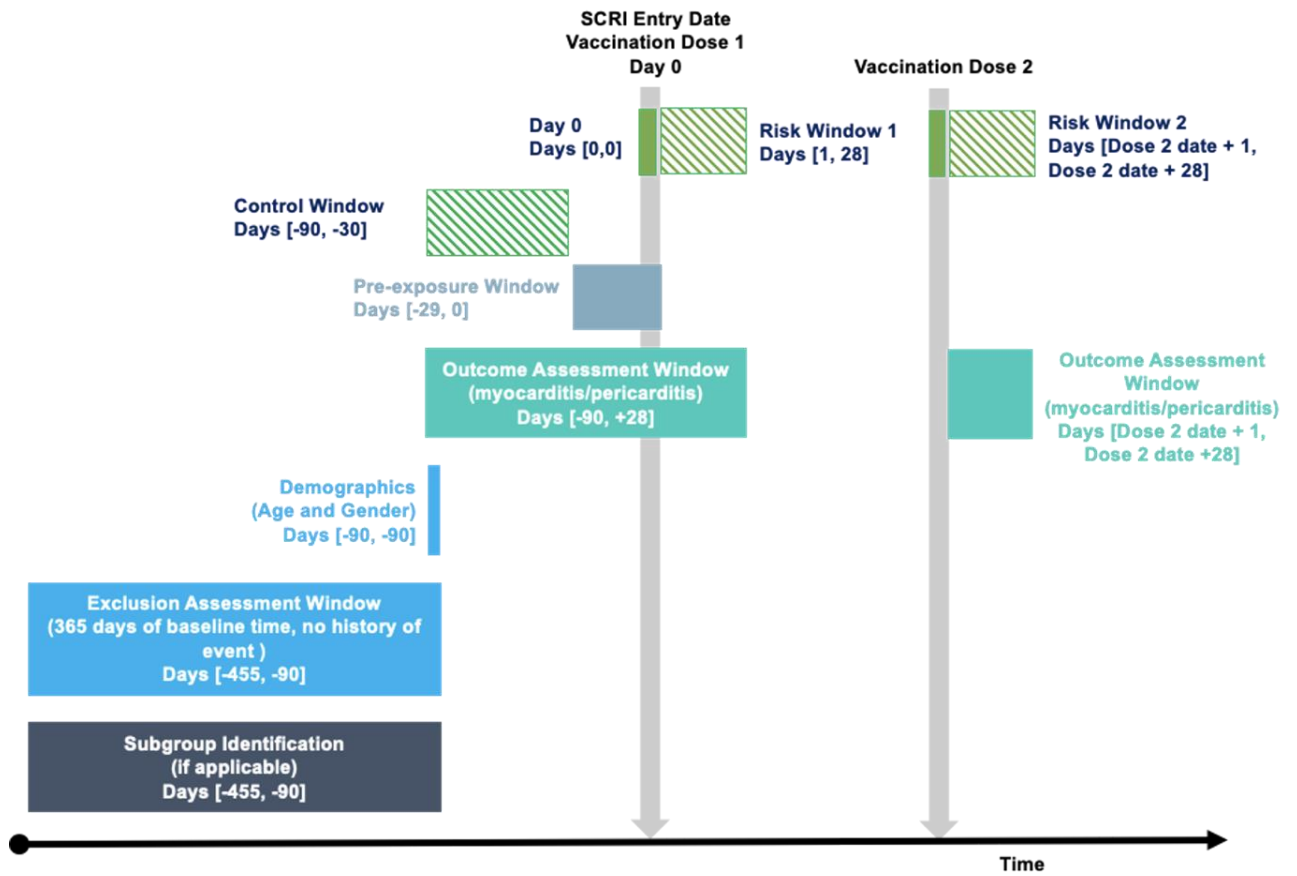

Supplement: Supplementary file 12 [file Image1.pdf]
