# Supplementary material for: Myocarditis and pericarditis associated with SARS-CoV-2 vaccines: A population-based descriptive cohort and a nested self-controlled risk interval study using electronic health care data from four European countries
Source: Front Pharmacol. 2022 Nov 24;13:1038043. doi: 10.3389/fphar.2022.1038043 (PMC9730238; doi:10.3389/fphar.2022.1038043)
Supplement: Supplementary file 4 [file Table9.DOCX]

Supplementary Table 4a. Myo- and pericarditis background incidence rate and rate differences per 100,000 person-years for persons aged <30 years by COVID-19 vaccine brand and dose

| Vaccine | | Age category | | Dose | Estimates* | IT-ARS | ES-BIFAP | ES-BIFAP-HOSP^&^ | ES-SIDIAP | UK-CPRD |
| --- | --- | --- | --- | --- | --- | --- | --- | --- | --- | --- |
| *Myocarditis* | | | | | | | | | | |
| None (*reference period*) | | 5 to 11 years | |  | Background IR | 2.9 [0.6 to 5.1] | 0.6 [0.1 to 1.1] | 1.1 [0.3 to 1.9] | 0.8 [0.1 to 1.7] | 0.5 [0.1 to 0.9] |
|  |  | 12 to 17 years | |  | Background IR | 9.9 [5.3 to 14.4] | 1.8 [0.8 to 2.8] | 2.9 [1.5 to 4.4] | 2.4 [0.1 to 4.1] | 1.2 [0.5 to 2.0] |
|  |  | 18 to 29 years | |  | Background IR | 6.4 [3.8 to 9.0] | 4.2 [3.1 to 5.3] | 5.6 [4.2 to 7.1] | 2.8 [1.5 to 4.1] | 4.1 [3.2 to 5.1] |
| Pfizer | | 5 to 11 years | | 1st | RD | - | - | - | - | - |
|  |  |  |  |  | Cases (PY) | 0 (650.5) | 0 (2537.8) | 0 (402.3) | 0 (5.6) | 0 (7040.6) |
|  |  |  |  | 2nd | RD | - | - | - | - | - |
|  |  |  |  |  | Cases (PY) | 0 (8.6) | 0 (3097.8) | 0 (517.5) | 0 (0.1) | 0 (77.6) |
|  |  | 12 to 17 years | | 1st | RD | - | - | - | 412.0 [-400.3 to 1224.3] | 8.7 [-2.5 to 19.9] |
|  |  |  |  |  | Cases (PY) | 0 (5222.5) | 0 (7484.0) | 0 (1157.7) | 1 (241.3) | <5 (30334.8) |
|  |  |  |  | 2nd | RD | 71.3 [-41.3 to 183.9] | 9.2 [-12.3 to 30.6] | 64.9 [-68.1 to 197.8] | - | 8.6 [-10.6 to 27.7] |
|  |  |  |  |  | Cases (PY) | <5 (2464.1) | <5 (9132.3) | 1 (1474.6) | 0 (172.6) | <5 (10239.1) |
|  |  | 18 to 29 years | | 1st | RD | 2.3 [-14.8 to 19.3] | - | - | - | -0.3 [-5.7 to 5.2] |
|  |  |  |  |  | Cases (PY) | <5 (11623.2) | 0 (13156.6) | 0 (1934.2) | 0 (2515.5) | <5 (52020.3) |
|  |  |  |  | 2nd | RD | 35.9 [-12.0 to 83.8] | 8.6 [-9.1 to 26.4] | - | - | 5.1 [-4.0 to 14.1] |
|  |  |  |  |  | Cases (PY) | <5 (7097.7) | <5 (15615.6) | 0 (2430.5) | 0 (2550.7) | <5 (43736.0) |
| Moderna | | 5 to 11 years | | 1st | RD | - | - | - | - | - |
|  |  |  |  |  | Cases (PY) | 0 (262.4) | 0 (397.9) | 0 (159.9) | 0 (0.1) | 0 (0.3) |
|  |  |  |  | 2nd | RD | - | - | - | - | - |
|  |  |  |  |  | Cases (PY) | 0 (0.4) | 0 (363.6) | 0 (152.8) | 0 (0.1) | 0 (0.2) |
|  |  | 12 to 17 years | | 1st | RD | - | 46.4 [-48.1 to 140.8] | - | - | - |
|  |  |  |  |  | Cases (PY) | 0 (1020.4) | <5 (2075.6) | 0 (393.8) | 0 (92.8) | 0 (928.8) |
|  |  |  |  | 2nd | RD | - | - | - | - | 406.3 [-54.8 to 867.3] |
|  |  |  |  |  | Cases (PY) | 0 (143.6) | 0 (1932.8) | 0 (375.4) | 0 (61.1) | <5 (736.3) |
|  |  | 18 to 29 years | | 1st | RD | - | - | - | - | 48.6 [-11.1 to 108.2] |
|  |  |  |  |  | Cases (PY) | 0 (4209.8) | 0 (5654.5) | 0 (891.1) | 0 (976.3) | <5 (5694.2) |
|  |  |  |  | 2nd | RD | **284.7 [29.6 to 539.9]** | 34.9 [-19.2 to 89.0] | 113.4 [-119.9 to 346.6] | - | - |
|  |  |  |  |  | Cases (PY) | 5 (1717.8) | <5 (5123.5) | 1 (840.3) | 0 (708.4) | 0 (4758.6) |
| AstraZeneca | | 5 to 11 years | | 1st | RD | - | - | - | - | - |
|  |  |  |  |  | Cases (PY) | 0 (0.1) | 0 (0.3) | NA | 0 (0.1) | 0 (2.4) |
|  |  |  |  | 2nd | RD | - | - | - | - | - |
|  |  |  |  |  | Cases (PY) | NA | NA | NA | NA | 0 (1.2) |
|  |  | 12 to 17 years | | 1st | RD | - | - | - | - | - |
|  |  |  |  |  | Cases (PY) | 0 (5.4) | 0 (109.6) | 0 (0.4) | 0 (54.8) | 0 (1092.7) |
|  |  |  |  | 2nd | RD | - | - | - | - | - |
|  |  |  |  |  | Cases (PY) | 0 (1.6) | 0 (62.3) | 0 (0.2) | 0 (30.7) | 0 (992.4) |
|  |  | 18 to 29 years | | 1st | RD | - | - | - | - | 3.0 [-10.9 to 16.9] |
|  |  |  |  |  | Cases (PY) | 0 (673.1) | 0 (3464.0) | 0 (66.4) | 0 (2203.8) | <5 (14147.1) |
|  |  |  |  | 2nd | RD | - | - | - | - | 11.5 [-10.2 to 33.2] |
|  |  |  |  |  | Cases (PY) | 0 (549.5) | 0 (2238.8) | 0 (57.7) | 0 (1527.1) | <5 (12807.5) |
| Janssen | | 5 to 11 years | | 1st | RD | - | - | - | - | - |
|  |  |  |  |  | Cases (PY) | NA | 0 (0.1) | NA | 0 (0.1) | NA |
|  |  | 12 to 17 years | | 1st | RD | - | - | - | - | - |
|  |  |  |  |  | Cases (PY) | 0 (0.8) | 0 (50.0) | 0 (0.8) | 0 (11.2) | 0 (0.5) |
|  |  | 18 to 29 years | | 1st | RD | - | - | - | - | - |
|  |  |  |  |  | Cases (PY) | 0 (12.9) | 0 (580.2) | 0 (16.0) | 0 (164.9) | 0 (5.3) |
| ***Pericarditis*** | | | | | | | | | | |
| None *(reference group)* | 5 to 11 years | |  | | Background IR | 5.2 [2.1 to 8.3] | 0.71 [0.2 to 1.3] | 0.9 [0.2 to 1.7] | 1.6 [0.3 to 2.8] | 0.6 [0.1 to 1.1] |
|  | 12 to 17 years | |  | | Background IR | 12.6 [7.5 to 17.8] | 6.2 [4.4 to 8.1] | 7.4 [5.1 to 9.6] | 10.8 [7.3 to 14.4] | 2.8 [1.7 to 3.9] |
|  | 18 to 29 years | |  | | Background IR | 10.0 [6.7 to 13.2] | 15.1 [13.0 to 17.2] | 18.8 [16.2 to 21.5] | 21.9 [18.2 to 25.5] | 10.3 [8.8 to 11.8] |
| Pfizer | 5 to 11 years | | | 1st | RD | - | - | - | - | 13.6 [-14.2 to 41.5] |
|  |  |  |  |  | Cases (PY) | 0 (650.6) | 0 (2537.7) | 0 (402.3) | 0 (5.6) | <5 (7040.4) |
|  |  |  |  | 2nd | RD | - | - | - | - | - |
|  |  |  |  |  | Cases (PY) | 0 (8.6) | 0 (3097.7) | 0 (517.5) | 0 (0.1) | 0 (77.6) |
|  | 12 to 17 years | | | 1st | RD | 25.7 [-27.6 to 79.0] | 7.1 [-19.1 to 33.4] | - | 404.1 [-409.2 to 1217.4] | 3.8 [-5.4 to 13.0] |
|  |  |  |  |  | Cases (PY) | <5 (5221.4) | <5 (7482.9) | 0 (1157.6) | <5 (241.0) | < 5 (30333.7) |
|  |  |  |  | 2nd | RD | 68.6 [-44.1 to 181.2] | 4.7 [-16.8 to 26.3] | 60.5 [-72.5 to 193.4] | - | 36.3 [-2.0 to 74.6] |
|  |  |  |  |  | Cases (PY) | <5 (2463.6) | <5 (9131.1) | <5 (1474.5) | 0 (172.4) | < 5 (10238.1) |
|  | 18 to 29 years | | | 1st | RD | 24.5 [-9.4 to 58.4] | 0.1 [-21.1 to 21.3] | 32.9 [-68.5 to 134.3] | 57.7 [-52.6 to 168.0] | 8.9 [-3.1 to 20.9] |
|  |  |  |  |  | Cases (PY) | <5 (11622.6) | <5 (13150.7) | <5 (1933.3) | < 5 (2514.4) | 10 (52011.2) |
|  |  |  |  | 2nd | RD | 18.2 [-21.0 to 57.4] | 29.8 [-3.5 to 63.1] | 22.3 [-58.4 to 103.1] | 17.4 [-59.6 to 94.3] | 1.1 [-9.0 to 11.3] |
|  |  |  |  |  | Cases (PY) | <5 (7097.6) | 7 (15608.5) | <5 (2429.3) | < 5 (2549.6) | 5 (43728.3) |
| Moderna | 5 to 11 years | | | 1st | RD | - | - | - | - | - |
|  |  |  |  |  | Cases (PY) | 0 (262.3) | 0 (397.9) | 0 (159.9) | 0 (0.1) | 0 (0.3) |
|  |  |  |  | 2nd | RD | - | - | - | - | - |
|  |  |  |  |  | Cases (PY) | 0 (0.4) | 0 (363.6) | 0 (152.8) | 0 (0.1) | 0 (0.2) |
|  | 12 to 17 years | | | 1st | RD | - | 90.2 [-43.4 to 223.8] | - | - | 104.9 [-106.2 to 316.0] |
|  |  |  |  |  | Cases (PY) | 0 (1020.1) | <5 (2074.9) | 0 (393.6) | 0 (92.6) | <5 (928.5) |
|  |  |  |  | 2nd | RD | - | - | - | - | - |
|  |  |  |  |  | Cases (PY) | 0 (143.5) | 0 (1932.2) | 0 (375.3) | 0 (61.0) | 0 (736.3) |
|  | 18 to 29 years | | | 1st | RD | 13.8 [-32.9 to 60.5] | 2.6 [-32.2 to 37.3] | - | 183.1 [-100.9 to 467.1] | 7.3 [-27.2 to 41.7] |
|  |  |  |  |  | Cases (PY) | <5 (4208.7) | <5 (5652.1) | 0 (890.8) | <5 (975.9) | <5 (5693.5) |
|  |  |  |  | 2nd | RD | 223.0 [-5.3 to 451.3] | 4.4 [-33.9 to 42.8] | 100.2 [-133.1 to 333.5] | - | - |
|  |  |  |  |  | Cases (PY) | <5(1717.3) | <5 (5121.3) | <5 (840.2) | 0 (708.0) | 0 (4758.1) |
| AstraZeneca | 5 to 11 years | | | 1st | RD | - | - | - | - | - |
|  |  |  |  |  | Cases (PY) | 0 (0.1) | 0 (0.3) | NA | 0 (0.1) | 0 (2.4) |
|  |  |  |  | 2nd | RD | - | - | - | - | - |
|  |  |  |  |  | Cases (PY) | NA | NA | NA | NA | 0 (1.2) |
|  | 12 to 17 years | | | 1st | RD | - | - | - | - | - |
|  |  |  |  |  | Cases (PY) | 0 (5.3) | 0 (109.4) | 0 (0.4) | 0 (54.8) | 0 (1092.3) |
|  |  |  |  | 2nd | RD | - | - | - | - | - |
|  |  |  |  |  | Cases (PY) | 0 (1.6) | 0 (62.3) | 0 (0.2) | 0 (30.6) | 0 (992.0) |
|  | 18 to 29 years | | | 1st | RD | - | 13.8 [-42.9 to 70.4] | - | - | -3.2 [-17.2 to 10.7] |
|  |  |  |  |  | Cases (PY) | 0 (673.2) | <5 (3463.2) | 0 (66.3) | 0 (2203.0) | <5 (14142.8) |
|  |  |  |  | 2nd | RD | - | - | - | - | 5.3 [-16.4 to 27.0] |
|  |  |  |  |  | Cases (PY) | 0 (549.6) | 0 (2238.2) | 0 (57.6) | 0 (1526.7) | <5 (12803.8) |
| Janssen | 5 to 11 years | | | 1st | RD | - | - | - | - | - |
|  |  |  |  |  | Cases (PY) | NA | 0 (0.1) | NA | 0 (0.1) | NA |
|  | 12 to 17 years | | | 1st | RD | - | - | - | - | - |
|  |  |  |  |  | Cases (PY) | 0 (0.8) | 0 (50.0) | 0 (0.8) | 0 (11.2) | 0 (0.5) |
|  | 18 to 29 years | | | 1st | RD | - | - | - | - | - |
|  |  |  |  |  | Cases (PY) | 0 (12.9) | 0 (579.9) | 0 (16.0) | 0 (164.5) | 0 (5.3) |

*IR: incidence rate; NA: not applicable; PY: person years; RD: rate difference*

^&^ ES-BIFAP-HOSP is a subpopulation of ES-BIFAP
