# Supplementary material for: Myocarditis and pericarditis associated with SARS-CoV-2 vaccines: A population-based descriptive cohort and a nested self-controlled risk interval study using electronic health care data from four European countries
Source: Front Pharmacol. 2022 Nov 24;13:1038043. doi: 10.3389/fphar.2022.1038043 (PMC9730238; doi:10.3389/fphar.2022.1038043)
Supplement: Supplementary file 5 [file Image3.pdf]

NNH and Excess events for risk period

Svetlana Belitser

3-6-2022

The first formula's for the attributable rate AR and the number needed to harm NNH are

from book "Self-Controlled Case Series Studies A Modelling Guide with R" Paddy Farrington, Heather Whitaker, Yonas Ghebremichael Weldeselassie

or

from article "Drug safety studies and measures of effect using the self-controlled case series design" Kumanan Wilson and Steven Hawken:

$$AR = \frac{\rho - 1}{\rho}\frac{n_1}{E} = \left(1 - \frac{1}{\rho}\right)\frac{n_1}{E}$$

$$NNH = \frac{1}{AR} = \frac{1}{1 - \frac{1}{\rho}}\frac{E}{n_1} \approx \frac{1}{1 - \hat{\rho}^{-1}}\frac{E}{n_1} = \frac{1}{1 - RR^{-1}_{Poisson\ regression}}\frac{E}{n_1}$$

incidence rate in control period IR_0 and in risk period IR_1:

$$IR_0 = \frac{n_0}{61\,E} = \frac{IR_{0;\ per\ PY\ per\ 10^5}}{365.25 * 10^5}; \quad IR_1 = \frac{n_1}{28\,E} = \frac{IR_{1;\ per\ PY\ per\ 10^5}}{365.25 * 10^5}$$

$$\Rightarrow E = \frac{n_0}{61\,IR_0} = \frac{n_1}{28\,IR_1}$$

$$\Rightarrow \quad \frac{E}{n1} == \frac{1}{n_1}\frac{n_0}{61\,IR_0} = \frac{\frac{n_0}{61}}{\frac{n_1}{28}28IR_0} = \frac{1}{28\,RR\,IR_0}$$

$$\Rightarrow NNH = \frac{1}{1 - \frac{1}{RR}}\frac{E}{n1} = \frac{1}{1 - \frac{1}{RR}}\frac{1}{28\,RR\,IR_0} = \frac{1}{RR - 1}\frac{1}{28\,IR_0} == \frac{1}{28}\frac{1}{RR - 1}\frac{365.25 * 10^5}{IR_{0;\ per\ PY\ per\ 10^5}}$$

$$AR = 28\,(RR - 1)\,IR_0 = 28\,(RR - 1)\,\frac{IR_{0;\ per\ PY\ per\ 10^5}}{365.25 * 10^5}$$

excess events for risk period [1;28days] per million vaccinated:

$$n_{excess\ events\ per\ 10^6} = AR * 10^6$$
