# Supplementary material for: Myocarditis and pericarditis associated with SARS-CoV-2 vaccines: A population-based descriptive cohort and a nested self-controlled risk interval study using electronic health care data from four European countries
Source: Front Pharmacol. 2022 Nov 24;13:1038043. doi: 10.3389/fphar.2022.1038043 (PMC9730238; doi:10.3389/fphar.2022.1038043)
Supplement: Supplementary file 6 [file Table5.DOCX]

**Supplementary Table 1**. Attrition diagram of participating data sources

|  | IT-ARS | ES-BIFAP | ES-BIFAP-HOSP | ES-SIDIAP | NL-PHARMO | UK-CPRD |
| --- | --- | --- | --- | --- | --- | --- |
| Persons in the instance of the data source | 4,062,263 | 16,271,838 | 16,271,838 | 6,968,416 | 2,401,054 | 15,993,226 |
| Sex or birth date missing or absurd, no dates of entry or exit | 0 | 29 | 29 | 545,955 | 0 | 0 |
| Death before study start | 131,491 | 1,005,204 | 1,005,204 | 268,269 | 88 | 0 |
| Exit from the data source before study start | 399,438 | 2,955,224 | 5,818,064 | 743,748 | 60,332 | 2,557,972 |
| Persons in the data source at study start | 3,531,334 | 12,311,381 | 9,448,541 | 5,410,444 | 2,340,634 | 13,435,254 |
| Less than 365 days history at 1//1/2020 | 40,959 | 314,692 | 236,634 | 126,500 | 38,616 | 1,138,611 |
| *Study population* | **3,490,375** | **11,996,689** | **9,211,907** | **5,283,944** | **2,302,018** | **12,296,643** |
