# Supplementary material for: Myocarditis and pericarditis associated with SARS-CoV-2 vaccines: A population-based descriptive cohort and a nested self-controlled risk interval study using electronic health care data from four European countries
Source: Front Pharmacol. 2022 Nov 24;13:1038043. doi: 10.3389/fphar.2022.1038043 (PMC9730238; doi:10.3389/fphar.2022.1038043)
Supplement: Supplementary file 7 [file Table13.DOCX]

Supplementary table 6: Pooled Incidence rate ratios of myocarditis and pericarditis in the whole population for the first and second vaccine dose per vaccine brand using 7-day risk windows

|  | **Myocarditis** | | **Pericarditis** | |
| --- | --- | --- | --- | --- |
|  | **First dose**  **IRR (95% CI)** | **Second dose**  **IRR (95% CI)** | **First dose**  **IRR (95% CI)** | **Second dose**  **IRR (95% CI)** |
| AstraZeneca  control window  1-7 days  8-14 days  15-21 days  22-28 days | *reference*  0.73 (0.16-3.26)  1.38 (0.43-4.41)  1.00 (0.11-9.25)  1.19 (0.33-4.37) | *reference*  1.52 (0.38-6.11)  3.63 (1.19-11.1)  1.06 (0.21-5.41)  2.64 (0.76-9.13) | *reference*  1.01 (0.50-2.06)  1.11 (0.12-19.7)  1.04 (0.61-1.80)  0.84 (0.46-1.51) | *reference*  0.99 (0.45-1.70)  1.26 (0.57-3.38)  1.20 (0.63-2.31)  1.52 (0.50-4.64) |
| Pfizer  control window  1-7 days  8-14 days  15-21 days  22-28 days | *reference*  3.55 (1.60-7.87)  2.05 (0.89-4.74)  1.53 (0.56-4.22)  1.42 (0.44-4.66) | *reference*  5.87 (2.71-12.8)  2.74 (1.05-7.14)  2.96 (1.11-7.86)  2.39 (0.87-6.57) | *reference*  0.83 (0.56-1.22)  0.87 (0.59-1.29)  1.21 (0.83-1.76)  0.83 (0.46-1.51) | *reference*  0.62 (0.37-1.03)  1.08 (0.70-1.65)  0.96 (0.61-1.51)  0.98 (0.62-1.55) |
| Moderna  control window  1-7 days  8-14 days  15-21 days  22-28 days | *reference*  11.8 (1.12-124.3)  6.39 (0.94-43.2) | *reference*  33.30 (7.55-146.7)  2.96 (0.10 -88.8)  22.10 (1.66-295.2) | *reference*  1.61 (0.65-3.98)  0.80 (0.28-2.31)  0.78 (0.30-2.02)  1.59 (0.43-5.38) | *reference*  2.49 (0.89-6.96)  0.60 (0.14-2.62)  0.92 (0.20-4.19)  1.13 (0.25-5.01) |
| Janssen  control window  1-7 days  8-14 days  15-21 days  22-28 days | *reference*  5.70 (0.42-77.3) |  | *reference*  3.38 (0.65-17.6)  2.09 (0.32-13.5) |  |

CI: confidence interval, IRR: incidence rate ratio. IRRs are adjusted for calendar time using 30-day periods
