# Supplementary material for: Myocarditis and pericarditis associated with SARS-CoV-2 vaccines: A population-based descriptive cohort and a nested self-controlled risk interval study using electronic health care data from four European countries
Source: Front Pharmacol. 2022 Nov 24;13:1038043. doi: 10.3389/fphar.2022.1038043 (PMC9730238; doi:10.3389/fphar.2022.1038043)
Supplement: Supplementary file 8 [file Table11.DOCX]

**Table 4c.** Myopericarditis cases by person-years and rate differences (directly standardised to Eurostat population for all persons above 30 years of age) per 100,000 PY by dose of vaccine and age category, with 95%CI in PHARMO among persons without recorded COVID-19

| Vaccine | Age category | Dose | Estimates* | NL-PHARMO |
| --- | --- | --- | --- | --- |
| *< 30 years* | | | | |
| None *(reference period)* | 12 to 17 years |  | Background IR | 2.6 [0.1 to 5.1] |
|  | 18 to 29 years |  | Background IR | 9.7 [6.3 to 13.1] |
|  |  |  |  |  |
| Pfizer | 12 to 17 years | 1st | Cases (PY) | 0 (4913.5) |
|  |  | 2nd | Cases (PY) | 0 (157.9) |
|  | 18 to 29 years | 1st | Cases (PY) | 0 (7555.0) |
|  |  | 2nd | Cases (PY) | 0 (864.6) |
| Moderna | 12 to 17 years | 1st | Cases (PY) | 0 (219.9) |
|  |  | 2nd | Cases (PY) | 0 (16.6) |
|  | 18 to 29 years | 1st | Cases (PY) | 0 (1149.1) |
|  |  | 2nd | RD | 733.36 [-722.94 to 2189.65] |
|  |  |  | Cases (PY) | <5 (134.6) |
| AstraZeneca | 12 to 17 years | 1st | Cases (PY) | 0 (26.7) |
|  |  | 2nd | Cases (PY) | 0 (6.0) |
|  | 18 to 29 years | 1st | Cases (PY) | 0 (279.3) |
|  |  | 2nd | Cases (PY) | 0 (60.7) |
| Janssen | 12 to 17 years | 1st | Cases (PY) | 0 (166.8) |
|  | 18 to 29 years | 1st | Cases (PY) | 0 (735.9) |
| *> 30 years* | | | | |
| None *(reference period)* | | | Background IR | 21.2 [18.9 to 23.6] |
| Pfizer | | 1st | RD | -4.8 [-17.0 to 7.5] |
|  |  |  | Cases (PY) | 8 (45400.0) |
|  |  | 2nd | RD | 4.0 [-19.0 to 27.0] |
|  |  |  | Cases (PY) | 6 (20380.5) |
| Moderna | | 1st | Cases (PY) | 0 (4385.0) |
|  |  | 2nd | RD | 109.2 [-146.3 to 364.6] |
|  |  |  | Cases (PY) | <5 (1514.2) |
| AstraZeneca | | 1st | RD | -8.2 [-29.1 to 12.6] |
|  |  |  | Cases (PY) | <5 (9232.9) |
|  |  | 2nd | Cases (PY) | 0 (2949.2) |

*IR: incidence rate; NA: not applicable; PY: person years; RD: rate difference*

* Estimates above 30 years of age are age-standardised to the Eurostat population and provided in 100,000 person-years. When cases are greater than 0, rate differences against age specific (standardized) background rare provided.
