# Supplementary material for: Myocarditis and pericarditis associated with SARS-CoV-2 vaccines: A population-based descriptive cohort and a nested self-controlled risk interval study using electronic health care data from four European countries
Source: Front Pharmacol. 2022 Nov 24;13:1038043. doi: 10.3389/fphar.2022.1038043 (PMC9730238; doi:10.3389/fphar.2022.1038043)
Supplement: Supplementary file 9 [file Table6.DOCX]

Supplementary Table 1. Codelist definitions for myocarditis, pericarditis and myopericarditis

| Event | Dictionary | Codes |
| --- | --- | --- |
| Myocarditis | ICD9 | "422","422,91","422,93","422.0","422.9","422.90","422.99","429.0" |
|  | ICD10 | "I40","I41","I51.4","I40.1","I40.8","I40.9" |
|  | READ | "G52..","G520.","G520z","G52y.","G52y0","G52yz","G52z.","G5y0.","Gyu5F","Gyu5G","Gyu5H","Gyu5J","Gyu5K","Gyu5L","X779D","XaDyL","G52..","G520z","G52y.","G52y2","G52y7","G52yz","G5y0.","Gyu5F","Gyu5H","Gyu5J","Gyu5K","Gyu5L" |
|  | ICPC | "K70" |
|  | ICPC2P | "K70002" |
|  | SNOMED | "155336004","155380004","194942007","194951004","194952006","194953001","194954007","194955008","194960007","194961006","195119007","195568002","195569005","195570006","195571005","195572003","195573008","251060004","266238009","31993003","37925008","45093008","45124100000000000000","46701001","488007","50920009","91025000","11176009","155380004","194942007","194951004","194952006","194953001","194955008","194960007","194961006","195119007","195568002","195569005","195570006","195571005","195572003","195573008","22653005","251060004","266238009","31993003","45093008","46701001","488007","50920009","723863003","89141000","91025000" |
| Pericarditis | ICD9 | "420.90","420.99","423.2","420","420.90","420.99","423.2", "420.99","423","423.3","423.9" |
|  | ICD10 | "I30","I30.8","I30.9","I32","I32.0","I32.1","I32.8","A18.84","I30","I30.1","I30.8","I30.9","I31.9","I32", "I31","I31.9","I30.1","I31","I31.4","I31.9" |
|  | READ | “24C..","24CA.","A17y2","G5003","G50z0","G50z2","G50z3","G50z4","G50zz","G532.","G5321","G532z","G53yz","Gyu50","Gyu52","Gyu53","Gyu54", "G50z5","G53..","G53z." |
|  | ICPC | - |
|  | ICPC2 | "K70003", "K84010" |
|  | SNOMED | "D-7484","F-74750","M-40000","M-46000","140297001","140300006","14455008","155333007","155340008","15555002","163083006","163086003","173574009","194902002","194908003","194914005","194916007","194917003","194918008","194919000","194920006","194968000","194969008","194974000","195552008","195554009","195555005","195556006","19880000","23627006","2554006","266236008","301124004","3238004","39517008","5836005","67256000","7036007","7607008","85598007","140300006","14455008","15555002","163083006","163086003","173574009","17668000","177291008","194908003","194914005","194916007","194917003","194918008","194920006","194969008","195552008","195554009","195555005","195556006","233881003","233883000","23627006","2554006","266235007","266236008","301124004","3238004","37715009","39517008","40959008","41739008","5836005","67256000","70189005","7036007","7607008","81376009","85598007","86504008", "D-7480","F -70330","NOCODE","155337008","155341007","194919000","194962004","194975004","22167000","25503004","266240004","266295005","35304003","55855009","155337008","194962004","22167000","25503004","266240004","35304003","373945007","55855009" |
| Myopericarditis | ICD9 | "420","422","422.0","422.9","422.90","422.91","422.93","422.99","423.2","429.0", "423.9" |
|  | ICD10 | "I30","I32","I40","I41","I51.4","I40.1","I40.8","I40.9" |
|  | READ | "G50z0","G50zz","G52..","G520.","G520z","G52y.","G52y0","G52yz","G52z.","G532.","G532z","G5y0.","Gyu50","Gyu52","Gyu53","Gyu54","Gyu5F","Gyu5G","Gyu5H","Gyu5J","Gyu5K","Gyu5L","X201j","X779D","XE0Up","XaDyL","G50..","G52..","G520z","G52y.","G52y2","G52y7","G52yz","G5y0.","Gyu50","Gyu52","Gyu53","Gyu54","Gyu5F","Gyu5H","Gyu5J","Gyu5K","Gyu5L", "X201i","G53.." |
|  | ICPC | "K70" |
|  | ICPC2 | "K70002","K70003", "K84010" |
|  | SNOMED | "155333007","155336004","155340008","155380004","15555002","194902002","194903007","194905000","194906004","194907008","194908003","194909006","194910001","194914005","194916007","194917003","194918008","194920006","194942007","194951004","194952006","194953001","194954007","194955008","194960007","194961006","194969008","195119007","195552008","195554009","195555005","195556006","195568002","195569005","195570006","195571005","195572003","195573008","233883000","233885007","251060004","266235007","266238009","31993003","3238004","37715009","37925008","45093008","45124100000000000000","46701001","488007","50920009","70189005","85598007","91025000","11176009","155380004","15555002","17668000","177291008","194914005","194920006","194942007","194951004","194952006","194953001","194955008","194960007","194961006","194969008","195119007","195552008","195554009","195555005","195556006","195568002","195569005","195570006","195571005","195572003","195573008","22653005","233881003","251060004","266238009","31993003","3238004","40959008","41739008","45093008","46701001","488007","50920009","723863003","81376009","85598007","86504008","89141000","91025000", "155341007","194964003","194965002","194970009","22167000","23412002","23627006","266295005","35304003","373945007","37980001","391179008","42653000","55855009","57231008","60254004","842701000000106","85598007","55855009" |
