# Supplementary material for: Myocarditis and pericarditis associated with SARS-CoV-2 vaccines: A population-based descriptive cohort and a nested self-controlled risk interval study using electronic health care data from four European countries
Source: Front Pharmacol. 2022 Nov 24;13:1038043. doi: 10.3389/fphar.2022.1038043 (PMC9730238; doi:10.3389/fphar.2022.1038043)
Supplement: Supplementary file 10 [file Table14.DOCX]

Supplementary table 7: Pooled incidence rate ratios of myocarditis and pericarditis for the first and second vaccine dose per vaccine brand stratified by both age and sex

|  | **Myocarditis** | | | | **Pericarditis** | | | |
| --- | --- | --- | --- | --- | --- | --- | --- | --- |
|  | **Unexposed/exposed**  **cases** | **First dose**  **IRR (95% CI)** | **Unexposed/exposed**  **cases** | **Second dose**  **IRR (95% CI)** | **Unexposed/exposed**  **cases** | **First dose**  **IRR (95% CI)** | **Unexposed/exposed**  **cases** | **Second dose**  **IRR (95% CI)** |
| **Women**  *12-29 years old*  Control  Pfizer  AstraZeneca  Moderna  Janssen | <5/<5  <5/<5  <5/<5 | *reference*  2.15 (0.30-15.4) | <5/<5  <5/<5  <5/<5 | *reference*  2.69 (0.16-44.8) | 10/8  <5/<5  <5/<5 | *reference*  1.86 (0.58-6.01)  2.71 (0.54-13.5) | 9/<5  <5/<5  <5/<5 | *reference*  1.97 (0.53-7.41)  1.89 (0.12-30.6) |
| *> 30 years*  Control  Pfizer  AstraZeneca  Moderna  Janssen | 17/7  15/<5  <5/<5  <5/<5 | *reference*  0.77 (0.27-2.22)  0.16 (0.02-1.27) | 18/11  12/5  <5/<5 | *reference*  1.23 (0.39-3.90)  1.01 (0.33-3.09) | 152/41  57/22  28/5  11/<5 | *reference*  0.71 (0.47-1.09)  0.90 (0.39-2.09)  0.24 (0.07-0.80) | 130/39  43/20  27/8 | *reference*  0.65 (0.44-0.96)  1.26 (0.49-3.25)  0.82 (0.35-1.93) |
| **Men**  *12-29 years old*  Control  Pfizer  AstraZeneca  Moderna  Janssen | 7/7  7/<5  <5/<5 | *reference*  3.19 (0.78-13.2)  0.29 (0.03-2.47)  5.96 (0.61-58.1) | <5/12  6/<5  <5/9 | *reference*  8.69 (2.54-29.7)  0.34 (0.04-2.94)  4.63 (0.40-53.4) | 31/18  6/<5  9/8  <5/<5 | *reference*  1.60 (0.85-3.02)  1.60 (0.55-4.71) | 21/18  6/<5  7/5 | *reference*  2.00 (1.04-3.84)  1.48 (0.33-6.69)  0.48 (0.05-4.18) |
| *> 30 years*  Control  Pfizer  AstraZeneca  Moderna  Janssen | 25/11  15/9  8/<5  <5/<5 | *reference*  1.39 (0.63-3.04)  1.57 (0.63-3.93)  0.67 (0.05-8.43)  0.92 (0.07-11.8) | 23/19  11/10  6/<5 | *reference*  1.69 (0.85-3.36)  2.85 (1.09-7.40)  0.84 (0.14-5.12) | 198/59  77/33  29/11  9/<5 | *reference*  0.91 (0.65-1.29)  0.93 (0.61-1.43)  1.54 (0.71-3.30)  1.62 (0.32-8.16) | 163/57  68/31  22/8 | *reference*  0.90 (0.64-1.24)  1.05 (0.66-1.68)  1.06 (0.45-2.48) |

CI: confidence interval, IRR: incidence rate ratio. IRRs are adjusted for calendar time using 30-day periods
