# Supplementary material for: Myocarditis and pericarditis associated with SARS-CoV-2 vaccines: A population-based descriptive cohort and a nested self-controlled risk interval study using electronic health care data from four European countries
Source: Front Pharmacol. 2022 Nov 24;13:1038043. doi: 10.3389/fphar.2022.1038043 (PMC9730238; doi:10.3389/fphar.2022.1038043)
Supplement: Supplementary file 11 [file Table10.DOCX]

**Table 4b**. Myo- and pericarditis background incidence rate and rate differences per 100,000 person-years for persons aged ≥30 years by COVID-19 vaccine brand and dose

| Vaccine | Dose | Estimates* | IT-ARS | ES-BIFAP | ES-BIFAP-HOSP^&^ | ES-SIDIAP | UK-CPRD |
| --- | --- | --- | --- | --- | --- | --- | --- |
| *Myocarditis* | | | | | | | |
| None *(2020 reference period)* | | Background IR | 4.5 [3.7 to 5.5] | 2.7 [2.4 to 3.1] | 3.6 [3.1 to 4.1] | 4.1 [3.4 to 4.8] | 4.0 [3.6 to 4.5] |
| Pfizer | 1st | RD | 3.0 [-3.8 to 9.8] | -1.1 [-3.6 to 1.4] | 1.3 [-8.3 to 11.0] | -2.8 [-4.7 to -0.9] | 2.1 [-2.0 to 6.2] |
|  |  | Cases (PY) | 5 (89584.6) | <5 (199900.5) | <5 (17758.0) | <5 (94036.0) | 10 (170138.9) |
|  | 2nd | RD | 0.01 [-4.2 to 4.2] | 1.9 [-1.8 to 5.6] | 13.9 [-7.0 to 34.8] | 5.2 [-10.1 to 20.5] | 4.4 [-0.5 to 9.3] |
|  |  | Cases (PY) | 5 (90183.6) | 7 (212008.1) | <5 (23126.0) | <5 (81300.7) | 13 (164090.5) |
| Moderna | 1st | RD | - | 2.0 [-7.3 to 11.3] | - | - | 3.9 [-7.1 to 14.9] |
|  |  | Cases (PY) | 0 (17496.2) | <5 (32826.9) | 0 (3914.1) | 0 (15463.6) | <5 (8393.7) |
|  | 2nd | RD | 3.6 [-12.3 to 19.4] | - | - | 22.3 [-29.3 to 73.8] | 0.5 [-8.3 to 9.2] |
|  |  | Cases (PY) | <5 (12573.4) | 0 (26983.2) | 0 (3845.2) | <5 (8587.4) | <5 (7930.9) |
| AstraZeneca | 1st | RD | - | 2.7 [-6.0 to 11.4] | - | - | **-1.9 [-3.5 to -0.3]** |
|  |  | Cases (PY) | 0 (24304.5) | <5 (70753.9) | 0 (5711.1) | 0 (40146.8) | 7 (271692.3) |
|  | 2nd | RD | - | - | - | - | 1.9 [-1.6 to 5.4] |
|  |  | Cases (PY) | 0 (21916.5) | 0 (32037.7) | 0 (5558.1) | 0 (9794.8) | 14 (252191.2) |
| Janssen | 1st | RD | - | 9.58 [-14.55 to 33.71] | - | - | - |
|  |  | Cases (PY) | 0 (4967.1) | <5 (18161.8) | - | - | 0 (41.7) |
| ***Pericarditis*** | | | | | | | |
| None *(2020 reference period)* | | Background IR | 26.9 [25.0 to 29.0] | 16.1 [15.2 to 17.0] | 20.9 [19.8 to 22.1] | 29.7 [27.9 to 31.6] | 11.6 [10.9 to 12.4] |
| Pfizer | 1st | RD | -7.3 [-16.8 to 2.3] | -3.8 [-9.9 to 2.3] | 1.9 [-25.5 to 29.2] | -0.5 [-14.2 to 13.3] | 3.5 [-3.0 to 9.9] |
|  |  | Cases (PY) | 19 (89530.1) | 22 (199831.3) | <5 (17750.5) | 28 (93971.9) | 25 (170099.7) |
|  | 2nd | RD | -7.6 [-16.6 to 1.5] | **-5.9 [-11.0 to -0.9]** | 7.0 [-18.4 to 32.3] | -7.0 [-23.8 to 9.8] | 5.9 [-1.3 to 13.0] |
|  |  | Cases (PY) | 21 (90121.4) | 20 (211929.4) | 5 (23116.3) | 26 (81235.0) | 26 (164048.4) |
| Moderna | 1st | RD | **-22.2 [-31.7 to -12.7]** | 7.4 [-12.2 to 27.0] | 0.3 [-41.2 to 41.8] | -1.2 [-34.1 to 31.7] | 6.3 [-14.6 to 27.2] |
|  |  | Cases (PY) | <5 (17482.4) | 6 (32810.5) | <5 (3911.8) | <5 (15448.7) | <5 (8391.7) |
|  | 2nd | RD | 31.1 [-12.4 to 74.7] | 5.8 [-12.1 to 23.7] | - | 9.7 [-48.0 to 67.3] | - |
|  |  | Cases (PY) | 7 (12560.7) | 7 (26968.5) | 0 (3842.9) | <5 (8576.7) | 0 (7928.8) |
| AstraZeneca | 1st | RD | -**21.7 [-28.3 to -15.0]** | -0.7 [-13.3 to 11.9] | **-16.7 [-25.1 to -8.3]** | -**21.4 [-29.0 to -13.8]** | -0.8 [-5.3 to 3.6] |
|  |  | Cases (PY) | <5 (24295.1) | 15 (70730.9) | <5 (5708.9) | 7 (40124.3) | 28 (271640.2) |
|  | 2nd | RD | -11.4 [-32.2 to 9.4] | -7.7 [-18.9 to 3.5] | - | -16.5 [-35.2 to 2.2] | 5.2 [-0.7 to 11.1] |
|  |  | Cases (PY) | 5 (21907.6) | <5 (32027.7) | 0 (5555.7) | <5 (9789.7) | 40 (252139.4) |
| Janssen | 1st | RD | -6.7 [-35.9 to 22.6] | - | - | - | - |
|  |  | Cases (PY) | <5 (4964.6) | 0 (18156.7) | - | - | 0 (41.7) |

*IR: incidence rate; NA: not applicable; PY: person years; RD: rate difference*

*All estimates are age-standardised to the Eurostat population and expressed in 100,000 person-years. When cases are greater than 0, rate differences against age standardized background rates are calculated.

^&^ ES-BIFAP-HOSP is a subpopulation of ES-BIFAP
